# Phytochemical Analysis, Biological Activities, and Molecular Docking Studies of Root Extracts from *Paeonia* Species in Serbia

**DOI:** 10.3390/ph17040518

**Published:** 2024-04-17

**Authors:** Petar Batinić, Aleksandra Jovanović, Dejan Stojković, Gökhan Zengin, Ilija Cvijetić, Uroš Gašić, Natalija Čutović, Mirjana B. Pešić, Danijel D. Milinčić, Tamara Carević, Aleksandar Marinković, Branko Bugarski, Tatjana Marković

**Affiliations:** 1 Institute for Medicinal Plant Research “Dr Josif Pančić”, Tadeuša Košćuška 1, 11000 Belgrade, Serbia; ncutovic@mocbilja.rs (N.Č.); tmarkovic@mocbilja.rs (T.M.); 2 Institute for the Application of Nuclear Energy INEP, University of Belgrade, Banatska 31b, Zemun, 11080 Belgrade, Serbia; ajovanovic@inep.co.rs; 3 Institute for Biological Research “Siniša Stanković”, National Institute of the Republic of Serbia, University of Belgrade, Bulevar Despota Stefana 142, 11060 Belgrade, Serbia; dejanbio@ibiss.bg.ac.rs (D.S.); uros.gasic@ibiss.bg.ac.rs (U.G.); tamara.carevic@ibiss.bg.ac.rs (T.C.); 4 Science Faculty, Selcuk University, 42130 Konya, Turkey; gokhanzengin@selcuk.edu.tr; 5 Faculty of Chemistry, University of Belgrade, Students Square 10-13, 11000 Belgrade, Serbia; ilija@chem.bg.ac.rs; 6 Faculty of Agriculture, Institute of Food Technology and Biochemistry, University of Belgrade, Nemanjina 6, Zemun, 11080 Belgrade, Serbia; mpesic@agrif.bg.ac.rs (M.B.P.); danijel.milincic@agrif.bg.ac.rs (D.D.M.); 7 Faculty of Technology and Metallurgy, University of Belgrade, Karnegijeva 4, 11000 Belgrade, Serbia; marinko@tmf.bg.ac.rs (A.M.); branko@tmf.bg.ac.rs (B.B.)

**Keywords:** peony roots, chemical characterization, Fourier-transform infrared spectroscopy, antibacterial activity, enzyme-inhibitory activity, *in vitro* gastrointestinal digestion

## Abstract

Without being aware of their chemical composition, many cultures have used herbaceous peony roots for medicinal purposes. Modern phytopreparations intended for use in human therapy require specific knowledge about the chemistry of peony roots and their biological activities. In this study, ethanol–water extracts were prepared by maceration and microwave- and ultrasound-assisted extractions (MAE and UAE, respectively) in order to obtain bioactive molecules from the roots of *Paeonia tenuifolia* L., *Paeonia peregrina* Mill., and *Paeonia officinalis* L. wild growing in Serbia. Chemical characterization; polyphenol and flavonoid content; antioxidant, multianti-enzymatic, and antibacterial activities of extracts; and *in vitro* gastrointestinal digestion (GID) of hot water extracts were performed. The strongest anti-cholinesterase activity was observed in PT extracts. The highest anti-ABTS (2,2′-azino-bis(3-ethylbenzothiazoline-6-sulphonic acid) radical potential was observed in PP extracts, whereas against DPPH (2,2-diphenyl-1-picrylhydrazyl radicals), the best results were achieved with PO extracts. Regarding antibacterial activity, extracts were strongly potent against *Bacillus cereus.* A molecular docking simulation was conducted to gather insights into the binding affinity and interactions of polyphenols and other *Paeonia*-specific molecules in the active sites of tested enzymes. *In vitro* GID of *Paeonia* teas showed a different recovery and behavior of the individual bioactives, with an increased recovery of methyl gallate and digallate and a decreased recovery of paeoniflorin and its derivatives. PT (Gulenovci) and PP (Pirot) extracts obtained by UAE and M were more efficient in the majority of the bioactivity assays. This study represents an initial step toward the possible application of *Paeonia* root extracts in pharmacy, medicine, and food technologies.

## 1. Introduction

The genus *Paeonia* (fam. Paeoniaceae) consists of 32 species and is divided into three sections, which are well supported by morphological and molecular studies: (1) *Paeonia* L., (2) *Onaepia* Lindl (a herbaceous species that is distributed around the Mediterranean region and in the countries of the Far East), and (3) *Moutan* DC (a woody species predominantly growing on the South American continent) [1]. Section *Paeonia* L., which contains 22 species, has the widest distribution among the three sections, and the distribution center is in China. Also, it is known that the herbaceous peony flowers are significant ornamentally for Chinese people, while their roots have a variety of biological activities and effects shown in alternative medicine [2,3]. 

In recent years, the secondary metabolites of the herbaceous peonies have been intensively studied, and over 20 active compounds have been identified [2]. Monoterpene glycosides, phenol-carboxylic acids, flavonoids, and tannins are considered major organic groups of molecules in the root extracts of herbaceous peonies. Despite the use of herbaceous peonies for medicinal purposes, there is only one report of the contemporary age on the chemical analysis of their root extracts, and it was related to peony populations widespread in China [2]. On the other hand, water or water–ethanol extracts are one of the forms that make the use of herbal drugs, that is, bioactive molecules from medicinal plants, more suitable in conventional pharmacotherapy [4,5]. The researchers are looking for the most efficient extraction protocol that captures and preserves all of the bio-constituents while retaining their healthy and nutritional properties. Regarding that, the extraction protocols, which include liquid and solid phases, are rudely divided into traditional and novel (eco-friendly) methods, referring to Soxhlet, maceration (M), CO_2_-extraction, heat- and microwave-assisted extraction (MAE), and ultrasound-assisted extraction (UAE) [6,7,8]. Some of these procedures require a high consumption of toxic extraction medium and prolonged extraction time, making them non-economic and harmful to the environment. Therefore, recent investigations favor the use of MAE and UAE in the extraction of active molecules from herbal material [9]. In addition, there is a theory that high temperature and pressure guarantee high extractability of bioactive compounds from herbal material with a short extraction time and a satisfactory degree of preservation of active molecules responsible for various biological and pharmacological activities [10]. 

As species of the *Paeonia* genus have become more favored for their edible, medicinal, and ornamental properties, in the first 20 years of the 21st century, large-scale chemical investigations and biological and medicinal tests have been performed, focusing especially on their generative organs (e.g., flowers, leaves, and stems) used to treat various diseases, including female sterility, neurological and urogenital disorders, and trauma [11]. The roots of *Paeonia officinalis* (PO) have been used in the therapy of sluggish liver, hepatomegaly, and chronic hepatitis [12]. On the other hand, in Chinese, Indian, and Perso-Arabic ethnomedicine, the root of PO was used as an ingredient in different antioxidant (i.e., natural) concoctions [11]. From the toxicological point of view, the root and root bark of PO were safe for administration up to a dose of 0.2 g/kg body weight/per day [13]. The root of *Paeonia peregrina* (PP) was a source of specific “cage-like” monoterpene glucosides, condensed tannins, and mono(di)-saccharides (e.g., aldoses) [14]. Even though the biological activity of PP is still very poorly investigated and refers only to the chemistry and biological potential of roots, flowers, and petals [15,16], its use for the treatment of epilepsy, spasms, and hemorrhage is documented [14,15]. Regarding the roots of *Paeonia tenuifolia* (PT), in the available literature, there is no published data about their biological activity. 

Numerous studies have been carried out utilizing different methods to evaluate the antioxidant, antibacterial, and enzyme-inhibitory properties of medicinal plants of importance with regard to food and functional food products and human health [16,17]. For this purpose, the most common methods for *in vitro* determination of the antioxidant, antibacterial, and enzyme-inhibitory activities of peony root extract were employed [18]. The antioxidant activity suggests that extracts could be used to alleviate the effects of oxidative stress caused by factors from the external environment (free radicals, pollution, ultraviolet radiation, etc.), which can lead to hindrance of a variety of diseases [16,17]. The extract’s antibacterial activity demonstrates its capacity to prevent the growth of pathogen bacteria that are already present in the human gastrointestinal tract (GIT), as evidenced by its ability to reduce the presence of these bacteria in people with impaired immune systems or digestive disorders [17,18]. The theory of enzymatic inhibition uses a bulk-based, deterministic approach to quantitatively evaluate how inhibitors influence the progression of enzyme action. However, single-enzyme catalysis is intrinsically stochastic, which may result in significant discrepancies from traditional requirements [18]. Cholinesterase inhibitors serve as a strategy for the treatment of neurological disorders (Alzheimer’s and Parkinson’s diseases, AD and PD, respectively); amylase and glucosidase inhibitors act as anti-diabetes mellitus factors, while tyrosinase inhibitors can be useful for dermatological disorders linked to excessive amounts of melanin, which can lead to skin cancer [19]. 

Up until today, there have been no comprehensive reports on the composition and content of secondary metabolites in the roots of any wild herbaceous peony species distributed in Serbia. For that reason, this study aimed to obtain root extracts of PT, PP, and PO using M, UAE, and MAE and to investigate their phytochemical composition by employing Ultra High Performance Liquid Chromatography (UHPLC-LTQ-OrbiTrap MS), UV-Visible (UV-Vis), and Fourier-transform infrared (FTIR) analyses. Also, this study aimed to systematically evaluate their biological potential through antioxidant, antibacterial, and enzyme inhibitory assays. A molecular docking simulation was conducted to gather valuable insights into the binding affinity and interactions of polyphenols and other *Paeonia*-specific molecules in the active sites of tested enzymes (acetylcholinesterase (AChE), butyrylcholinesterase (BuChE), human pancreatic α-amylase (HPA), human intestinal α-glucosidase (HIG), and tyrosinase). Additionally, the aim was to evaluate the bioaccessibility of the bioactive compounds in hot water extracts (teas) and estimate their potential for use in the food industry. As a result of the overall research, the goal of this study was also to recommend the best plant resources for further cultivation that would enable the continuous supply of interested industries with herbal raw materials (herbaceous peony roots) of standardized quality. 

## 2. Results and Discussion

In the present study, PT, PP, and PO root extracts were prepared using M, UAE, and MAE. The obtained extracts were characterized via identification and determination of bioactives, *in vitro* estimation of biological activities, and *in vitro* release of biologically active compounds in simulated gastrointestinal fluids.

### 2.1. Chemical Characterization

**Figure 1:**
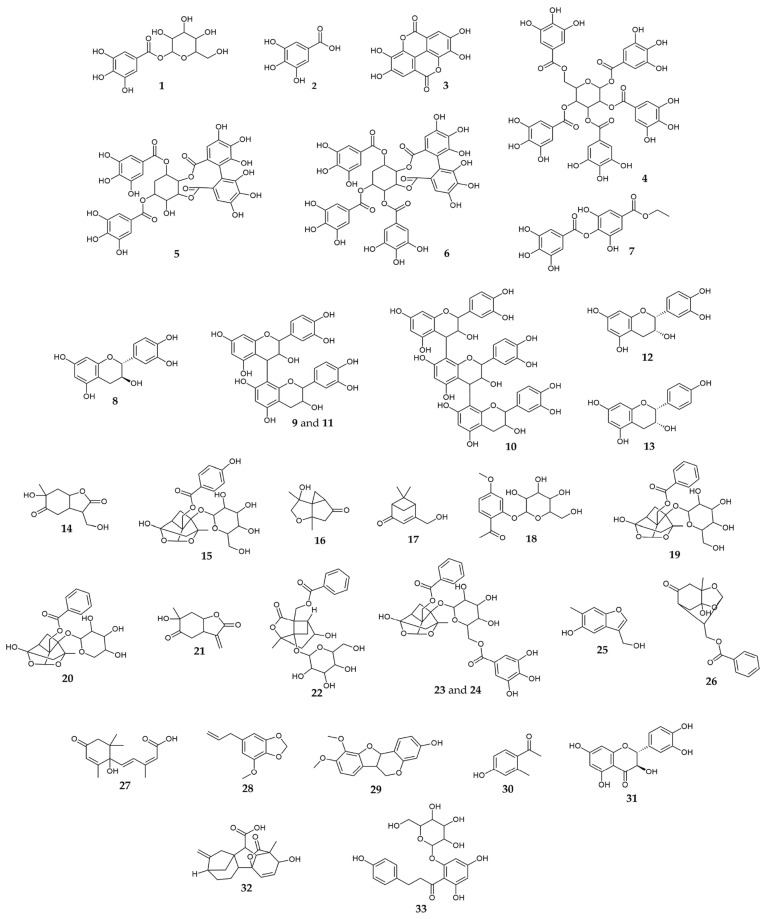
Chemical structures of major active compounds identified in the root extracts of *Paeonia tenuifolia* L., *Paeonia peregrina* Mill., and *Paeonia officinalis* L.

The chemical characterization of the roots of the wild peonies that grow in Serbia is summarized in Table 1. The extracts of roots PT, PP, and PO are a rich source of phenolic compounds, and four groups of polyphenols were identified: (1) gallic acid derivatives (compounds **1**–**7**), (2) flavan-3-ols (compounds **8**–**13**), (3) *Paeonia* terpenes (compounds **14**–**26**), and (4) other metabolites (compounds **27**–**33**). From the full-scan MS spectra, molecular formulas of the compounds were obtained using isotopic exact masses, while the exact structure was suggested by further study of the MS^2–4^ spectra. The chemical structures of major active compounds identified in PT, PP, and PO obtained by microwave-assisted extraction (MAE) are presented in Figure 1. All listed compounds in Table 1 have been previously isolated or identified in *Paeonia* taxa.

Gallic acid derivatives. This group of molecules presents the major peony antioxidants. The MS data of compounds **1, 2, 3,** and **7** matched with galloyl-hexose, gallic acid, ellagic acid, and ethyl-digallate, respectively [20,21,22,23]. Compound **4** eluted at 5.00 min showed a molecular ion at 939 *m*/*z,* and from its exact mass, the chemical formula was calculated–C_41_H_31_O_26_^−^. The MS^2^ base peak, formed by the neutral loss of one gallic acid molecule (170 Da), was found at 769 *m*/*z*. MS^3^ and MS^4^ base peaks were formed by subsequent loss of 152 Da, respectively, and fragments at 617 and 465 *m*/*z* were obtained. Finally, with all the abovementioned facts, this compound was identified as pentagalloyl-hexose, and its fragmentation agrees with the available literature [18]. Also, it is reported that pentagalloyl-hexose (**4**) is exclusively based on 1,2,3,4,6-pentagalloylglucose core, so that the depside of the galloyl group is mainly connected at the C-3 and C-4 positions of the glucose group through *m*- and *p*-deleterious linkages [24,25,26]. On the other hand, compounds **5** and **6**, derivatives of protoquercitol, have not been previously found in the chemical composition of the roots of plants belonging to the *Paeonia* taxa. Compounds **1, 2, 3, 4,** and **7** have previously been found to be the main constituents of the chemical composition of the plant species belonging to the *Paeonia* taxa [16,17]. 

Flavon-3-ols. Compounds **8, 12,** and **13** were identified as flavan-3-ol monomers (catechin, epicatechin, and epiafzelechin, respectively) by comparing their fragment ions with the reference standards in the online database [27,28,29]. The characteristic MS fragmentation pattern of catechin and epicatechin was previously described by Xu et al. [27]. Compound **12** was detected at 4.23 min with a molecular ion at 291 *m*/*z*. Its MS^2^ base peak found at 139 *m*/*z* was formed by characteristic retro-Diels-Alder (RDA) fragmentation and the loss of 152 Da—1,3 + RDA fragment [30]. The MS^3^ base peak was at 111 *m/z* (generated by a loss of CO—28 Da), while its MS^4^ base peak (83 *m*/*z*) was generated by a further loss of CO (28 Da). Compounds **9, 10,** and **11** showed molecular ions of 579, 867, and 579 *m*/*z,* respectively. These compounds could be tentatively identified as B-type procyanidin dimers 1 and 2 (compounds **9** and **11**) and B-type procyanidin trimer (compound **10**).

*Paeonia terpenes.* This group of compounds was the main chemical constituent of the extracts of PT, PP, and PO. Therefore, **12** compounds from this class were detected, and all of them were previously reported as characteristic of the *Paeonia* taxa [16]. Compounds **15** (*t*_R_ = 3.28 min) and **22** (*t*_R_ = 4.91 min), as well as their isomers (compounds **23**, *t*_R_ = 5.02) and **24**, *t*_R_ = 5.32) were identified as oxypaeoniflorin, albiflorin, galloyl-paeonoflorin (631 *m/z*), and galloyl-paeoniflorin isomer, respectively [31]. The typical paeoniflorin derivatives had the characteristic fragment ion [M−H_2_O−H]^−^, and for the pinane skeleton of a molecule of paeniflorin, it was easy to lose the neutral H−COH (30 Da), mainly due to the instability of the hemiketal group [27]. Oxypaeoniflorin had a negative molecular ion [M−H]^−^ of 495 *m*/*z*, and a prominent MS^2^ base peak fragment ion of 465 *m*/*z*. Also, several organic molecules identified in this study were previously reported in other herbaceous and tree peonies: compound **16** with molecular ion at 183 *m*/*z* was found in the cortex of a mountain peony (*Paeonia suffruticosa*) [32]; compound **17** with molecular ion at 167 *m*/*z* was found in the root of herbaceous *Paeonia lactiflora* [31]; and compound **18** (paenol glycoside) with molecular ion at 327 *m/z* was found both in the roots of *P. suffruticosa* and *P. lactiflora* [1]. Furthermore, the identification of compound **25**, precisely paeoveitol D, with a molecular ion at 179 *m*/*z*, was achieved by its characteristic MS fragmentations. Compound **26** (paeoniflorigenone), a new monoterpene that modulates the neuromuscular junction in mouse-isolated phrenic nerve-diagram preparation, was previously isolated from the roots of *Paeonia albiflora* Pall. [33] and from the root bark of *P. suffriticosa* [34]. 

Other metabolites. In this group, several specific organic compounds were identified, and some of them are strong repellents and plant hormones that regulate numerous aspects of plant growth, development, and stress responses. Abscisic acid (**27**) with a molecular ion at 265 *m*/*z* was first found in the leaves of *P. lactiflora* Pall., and it was responsible for the positive impact of leaf senescence [34]. Myristicin (**28**), with a molecular ion at 193 *m*/*z*, was a strong acaricide that inhibits monoamine oxidase responsible for the promotion of angiogenesis [34], and it was previously found in the essential oil of *Paeonia mascula* [35,36,37]. Compound **29** was previously detected in the dried root of *P. lactiflora* Pall. [38] and *Paeonia veitchii* Lynch, while compound **30** was found in the root-bark of *Paeonia ostii* [39]. Taxifolin (**31**) is a natural antioxidant that belongs to the subclass of flavanonols in the flavonoids, and it was found in the fruit extracts of the tree *Paeonia rockii* [40]. Compounds **32** and **33** (gibberellin and phloridzin) were found in the trees *P. ostii, P. rockii*, and *P. suffruticosa* [38,39,40]. 

**Table 1:** HRMS and MS^4^ data for secondary plant metabolites identified in the root extracts of *Paeonia tenuifolia* L. (PT), *Paeonia peregrina* Mill. (PP), and *Paeonia officinalis* L. (PO).

No	Compound Name	*t*_R_, min	Molecular Formula, [M ± H]^±^	Calculated Mass, *m*/*z*	Exact Mass, *m*/*z*	Δ ppm	MS^2^ Fragments, (% Base Peak)	MS^3^ Fragments, (% Base Peak)	MS^4^ Fragments, (% Base Peak)	PT	PP	PO	Refs.
Gallic acid derivatives													
1	Galloyl-hexose	0.59	C_13_H_15_O_10_^−^	331.06707	331.06588	3.61	125 (19), **169** (100), 193 (10), 211 (24), 271 (50)	**125** (100)	67 (58), 73 (59), **107** (100)	+	–	+	[19]
2	Gallic acid	0.91	C_7_H_5_O_5_^−^	169.01425	169.01355	4.14	**125** (100)	53 (57), 63 (50), **97** (100)	NA	+	+	+	[20]
3	Ellagic acid	4.62	C_14_H_5_O_8_^−^	300.99899	300.99744	5.16	185 (45), 201 (25), **229** (100), 257 (77), 284 (44)	129 (28), 157 (34), 173 (44), 185 (84), **201** (100)	NA	+	+	+	[15]
4	Pentagalloyl-hexose	5.00	C_41_H_31_O_26_^−^	939.11091	939.10833	2.74	617 (6), **769** (100), 787 (9)	429 (16), 447 (29), 599 (34), 601 (22), **617** (100)	193 (10), 313 (15), 429 (9), 447 (40), **465** (100)	+	–	+	[25]
5	Digalloyl-HHDP-protoquercitol	5.23	C_34_H_27_O_21_^+^	771.10394	771.10271	1.59	233 (29), 261 (75), 279 (50), **305** (100), 413 (14), 431 (60)	**153** (100)	**125** (100), 143 (25)	+	+	+	[24]
6	Trigalloyl-HHDP-protoquercitol	5.57	C_41_H_31_O_25_^+^	923.11489	923.11375	1.23	**305** (100), 431 (53), 457 (30), 601 (22), 771 (42)	**153** (100)	**125** (100), 143 (26)	+	+	+	[41]
7	Ethyl-digallate	5.93	C_16_H_13_O_9_^−^	349.05651	349.05455	5.61	**197** (100)	**125** (14), 169 (100)	**125** (100)	+	–	+	[21]
Flavan-3-ols													
8	Catechin	3.47	C_15_H_15_O_6_^+^	291.08632	291.08529	3.52	**123** (100), 139 (97), 147 (8), 151 (21), 165 (43), 273 (15)	67 (73), 77 (53), 95 (55), **105** (100), 199 (12)	NA	+	+	+	[25]
9	B-type procyanidin dimer 1	3.53	C_30_H_27_O_12_^+^	579.14970	579.14788	3.14	247 (28), 289 (33), 291 (66), 301 (20), 409 (60), **427** (100)	247 (14), 275 (62), 287 (18), 301 (69), **409** (100)	257 (20), **287** (100), 299 (10), 391 (11)	+	+	+	[42,43]
10	B-type procyanidin trimer	3.79	C_45_H_39_O_18_^+^	867.21309	867.2118	1.48	425 (19), 559 (20), 577 (88), **579** (100), 715 (19)	289 (23), 291 (48), 301 (27), 409 (75), **427** (100)	247 (23), 275 (43), 287 (8), 301 (43), **409** (100)	+	–	+	[42,43]
11	B-type procyanidin dimer 2	4.02	C_30_H_27_O_12_^+^	579.14970	579.14836	2.32	247 (23), 289 (38), 291 (71), 301 (17), 409 (37), **427** (100)	247 (19), 275 (67), 287 (14), 301 (68), **409** (100)	257 (19), **287** (100), 299 (7), 391 (11)	+	+	+	[42,43]
12	Epicatechin	4.23	C_15_H_15_O_6_^+^	291.08632	291.08547	2.9	123 (98), **139** (100), 147 (8), 151 (21), 165 (42), 273 (10)	67 (19), 83 (13), 93 (14), **111** (100)	65 (26), 69 (70), **83** (100), 93 (73), 111 (24)	+	+	+	[25]
13	Epiafzelechin	5.92	C_15_H_15_O_5_^+^	275.09140	275.09057	3.03	**107** (100), 127 (15), 149 (22), 169 (33)	53 (80), 77 (46), **79** (100), 93 (33), 99 (36)	NA	+	+	+	[26]
*Paeonia terpenes*													
14	9-Hydroxypaeonilactone A	2.42	C_10_H_15_O_5_^+^	215.09140	215.09072	3.16	**155** (100), 158 (26), 169 (19), 173 (14), 197 (81)	95 (26), 109 (42), 113 (26), 127 (28), **137** (100)	69 (47), 81 (25), 95 (39), 106 (7), **109** (100)	+	+	–	[29]
15	Oxypaeoniflorin	3.28	C_23_H_27_O_12_^−^	495.15080	495.14796	5.74	331 (20), 333 (22), 427 (32), 449 (27), **465** (100)	NA	NA	+	–	+	[44]
16	Paeonisothujone	3.71	C_10_H_15_O_3_^+^	183.10157	183.10085	3.96	137 (14), 147 (69), **165** (100)	123 (18), 137 (24), **147** (100)	**119** (100), 129 (20)	+	+	–	[30]
17	Paeonisuffrone C	3.94	C_10_H_15_O_2_^+^	167.10666	167.10622	2.63	109 (22), 121 (27), 125 (17), **149** (100)	93 (11), 107 (24), 109 (8), **121** (100), 131 (81)	64 (14), 77 (15), 91 (40), **93** (100), 105 (30)	+	+	+	[29]
18	Paeonoside	4.28	C_15_H_19_O_8_^−^	327.10854	327.10743	3.39	123 (14), **165** (100), 309 (12)	95 (29), 121 (19), **123** (100)	NA	+	–	–	[22]
19	Paeoniflorin + FA	4.39	C_24_H_29_O_13_^−^	525.16137	525.15874	5.00	**449** (100), 479 (33)	165 (42), 309 (8), **327** (100)	123 (10), **165** (100), 309 (16)	+	+	+	[44]
20	Paeoniflorigenin 1-O-pentoside	4.40	C_22_H_25_O_10_^−^	449.14532	449.14346	4.13	165 (25), 309 (3), **327** (100)	123 (15), **165** (100), 309 (28)	**123** (100), 147 (21), 150 (14)	+	+	+	[45]
21	Paeonilactone B	4.53	C_10_H_13_O_4_^+^	197.08084	197.07993	4.57	125 (41), **138** (100), 179 (20)	69 (13), 83 (7), **110** (100)	69 (94), **83** (100), 94 (30)	+	+	+	[25]
22	Albiflorin + FA	4.91	C_24_H_29_O_13_^−^	525.16137	525.15859	5.29	**449** (100), 479 (41)	165 (38), 309 (7), **327** (100)	123 (11), **165** (100), 309 (11)	+	–	+	[44,45]
23	Galloyl-paeoniflorin	5.02	C_30_H_31_O_15_^−^	631.16684	631.16434	3.96	271 (20), 313 (10), 399 (10), 479 (12), 491 (23), **613** (100)	**271** (100), 313 (33), 375 (16), 399 (27), 491 (67)	169 (8), 210 (7), **211** (100)	+	–	+	[44,45]
24	Galloyl-paeoniflorin isomer	5.32	C_30_H_31_O_15_^−^	631.16684	631.16451	3.70	**313** (100), 463 (9), 481 (25), 483 (6), 509 (12)	125 (37), 151 (31), **169** (100), 209 (11), 223 (12)	**125** (100)	+	–	+	[44,45]
25	Paeoveitol D	5.38	C_10_H_11_O_3_^+^	179.07027	179.06977	2.82	93 (21), 107 (32), 135 (97), **151** (100), 161 (14)	95 (21), 105 (19), **109** (100), 123 (80), 133 (31)	65 (3), 69 (10), 79 (6), **81** (100), 91 (14)	+	+	+	[46]
26	Paeoniflorigenone	8.13	C_17_H_19_O_6_^+^	319.11762	319.11663	3.07	179 (15), **197** (100), 259 (8), 273 (10), 301 (8)	119 (21), 137 (33), 155 (59), 161 (14), **179** (100)	85 (13), 133 (21), **137** (100), 151 (10), 161 (24)	+	+	+	[32]
Other metabolites													
27	Abscisic acid	3.80	C_15_H_21_O_4_^+^	265.14344	265.14258	3.22	175 (13), 203 (17), 229 (12), 235 (8), **247** (100)	157 (52), 187 (36), 201 (48), 217 (56), **229** (100)	159 (27), 173 (21), 187 (40), **201** (100), 211 (68)	–	+	+	[33]
28	Myristicin	3.96	C_11_H_13_O_3_^+^	193.08592	193.08535	2.98	138 (18), **161** (100), 166 (14)	105 (22), **133** (100)	**105** (100)	+	+	+	[35]
29	3-Hydroxy-9,10-dimethoxypterocarpan	4.53	C_17_H_17_O_5_^+^	301.10705	301.10602	3.40	187 (18), 243 (40), **283** (100), 285 (16)	211 (31), 235 (19), **239** (100), 247 (24), 265 (23)	**211** (100), 213 (3)	+	+	+	[36]
30	4-Hydroxy-2-methylacetophenone	4.58	C_9_H_11_O_2_^+^	151.07536	151.07499	2.40	95 (30), 105 (25), **109** (100), 123 (93), 133 (38)	69 (8), 79 (8), **81** (100), 91 (12)	NA	+	+	+	[37]
31	Taxifolin	4.80	C_15_H_13_O_7_^+^	305.06558	305.06445	3.69	153 (99), 195 (35), 259 (87), **287** (100)	105 (9), **259** (100), 269 (3)	149 (76), **231** (100), 241 (10)	+	+	+	[40]
32	Gibberellin A7	5.06	C_19_H_23_O_5_^+^	331.15400	331.15286	3.45	151 (35), 189 (18), 285 (13), 287 (47), **313** (100)	137 (8), **151** (100), 189 (76), 249 (10), 281 (28)	91 (40), 95 (20), **119** (100), 123 (4), 136 (8)	+	+	+	[39]
33	Phloridzin	5.66	C_21_H_23_O_10_^−^	435.12967	435.12705	6.03	205 (4), 271 (5), **273** (100), 293 (5), 399 (5)	123 (15), **167** (100)	**123** (100)	+	–	+	[47]

The **bold** numbers indicate 100% of the base peak, as well as which peaks were further fragmented in the MS^3^ and MS^4^ experiments; NA indicates not available. FA: formic acid; + stands for detected and – stands for not detected compounds; refs: the literature that indicates that the given compound was identified in *Paeonia*.

### 2.2. FTIR Study

**Figure 2:**
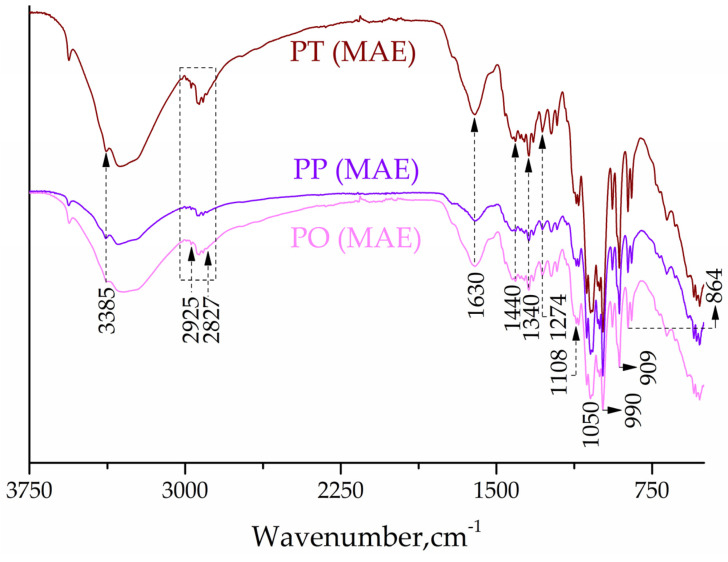
ATR-FTIR spectra of the root extracts of *Paeonia tenuifolia* L. (PT), *Paeonia peregrina* Mill. (PP), and *Paeonia officinalis* L. (PO). MAE: microwave-assisted extraction.

Structural characterization via FTIR with the attenuated total reflectance (ATR) technique was used to identify characteristic functional groups presented in the extracts of PT, PP, and PO. As shown in Figure 2, the *Paeonia* root extracts (obtained by MAE) exhibited characteristic peaks that can be attributed to stretching and bending vibrations in different areas, as follows: O–H stretching (a broad band of a hydroxyl group bonded directly to the aromatic (phenol) hydrocarbon group at 3385 cm^−1^ and C–O stretching at 1274 cm^−1^); C–H asymmetric and symmetric stretching (–CH_3_ group at 2925 cm^−1^; –CH_2_/methoxy/–CH_3_ at 2897 cm^−1^); C=O stretching: from the aromatic organic molecules in flavonols (ester functionalities) at 1630 cm^−1^; –CH_3_ bending (the bands at 1440 and 1340 cm^−1^); C–O–C stretching that can be attributed to the carbohydrate residues (strong bands in the region between 1150 and 1000 cm^−1^ that is characteristic for the C–O stretching of –C–O–C– glycosidic linkages), and –CH bending (from an aromatic compound, band at 864 cm^−1^) [48,49]. 

The results of the FTIR study are in accordance with others reported earlier [4,5]. The weak absorption bands in the region of 1000–860 cm^−1^ can be assigned to the α− and β−glycosidic linkages [48,49]. The FTIR spectra of PT, PP, and PO obtained by UAE and M were provided in the Supplementary Materials (Figures S1 and S2). 

### 2.3. Optimization of the Polyphenol Extraction Process from Paeonia Roots

The values of the polyphenol yield of all prepared *Paeonia* root extracts are shown in Table 2. The total phenol content (TPC) values in PT extracts were in the range of 31.14 to 99.64 mg GAE (gallic acid equivalents)/g, depending on the employed extraction procedure and locality. Furthermore, the polyphenol concentration was between 32.39 and 161.2 mg GAE/g in the PP samples, while the TPC of the PO extracts varied from 35.01 to 165.6 mg GAE/g.

*Preliminary screening of factor levels.* The influence of all levels of each factor (locality and extraction method) on the TPC was examined. The results are presented in the Supplementary Materials (Figure S3). Statistical significance among all factor levels was estimated on triplicate values through a one-way ANOVA and Duncan’s post hoc test (*p* < 0.05). Selected two levels of each factor with the highest polyphenol yield were further included in the experimental design (Table 3).

Locality represents an important parameter that significantly influenced the TPC in all tested roots of *Paeonia* species (Figure S3). Namely, the effect of the locality of PT follows the trend: Gulenovci and Deliblato sand > Bogovo gumno (Figure S3). In the case of PP: Pirot and Pančevo > Južni kučaj ≥ Krivi vir and Golina ≥ Bogovo gumno (Figure S3), whereas in PO: Božurna ≥ Rujevica (Figure S3). Namely, the chemical composition and quantity of the target compounds in the harvested plant matrix are significantly affected by the geographic location, climate, soil, seasonal variations, and altitude [16]. It can be an explanation for the presence of a significant difference between the TPC values of the extracts prepared using roots from various localities. Therefore, Gulenovci and Deliblato sand (for PT), Pirot and Pančevo (for PP), and Božurna and Rujevica (for PO) were included in further experimental design. As can be seen in Figure S3, UAE provided the PT extracts with the highest TPC values; however, there were no statistically significant differences between UAE and MAE. Additionally, the extracts prepared using M method possessed the lowest TPC, but there were no statistically significant differences between the M method and MAE. According to the literature data, UAE represents a good alternative extraction procedure in comparison to traditional techniques due to its high efficiency, lower price, simplicity, reduced extraction time and solvent consumption, and employment of a wide range of extraction solvents. Namely, ultrasound waves in the extraction solvent induce mechanical, thermal, and cavitation reactions causing the degradation of cell structures without significant changes in the structural and functional characteristics of most target compounds [50]. Higher contents of polyphenol components in extracts obtained in the UAE, apart from reduced extraction time in comparison to traditional extraction procedures, were shown in several publications [51,52,53]. MAE also provides numerous benefits, including a shorter extraction time, a reduced amount of extraction medium, and higher extraction efficiency due to the high vapor pressure of free water molecules in the plant cells and the breaking of the cell walls that provide a higher release of the compounds in the surrounding medium [8]. On the other hand, the M method as a traditional procedure possesses several disadvantages, such as low extraction yield and prolonged extraction time [8]. According to the literature, the high power of microwaves can ensure the degradation of cell walls and better diffusion of polyphenols into extraction surroundings in shorter extraction periods. However, prolonged extraction time and a higher yield of MAE do not mean a large quantity of target components, such as polyphenols, because microwaves cause the release of a large amount of ballast compounds, including lipids, proteins, and sugars [8]. MAE is a widely and successfully applied procedure for preparing polyphenol-rich extracts from different plant materials [54]. In the case of PT, the results showed that the M method provided significantly lower values of polyphenol yield in comparison to MAE (Figure S3), therefore, it was excluded for further experimental design. Regarding the TPC of PP extracts, the impact of the extraction procedure follows the trend: MAE > UAE and M methods (Figure S3). Since there was no statistically significant difference in the TPC between UAE and M method and the M method does not require an expensive device, UAE was excluded from further experimental design related to the PT samples. In the PO extracts, there was no statistically significant difference between all employed extraction procedures (Figure S3), thus MAE and M method were included in future experimental design (MAE due to very short extraction time, while M method due to simple operation). According to the results of one-way ANOVA and Duncan’s post hoc test and the highest TPC values, the following two levels of two parameters (locality and extraction method) were included in the experimental design (Table 3): for PP—Gulenovci and Deliblato (among localities), and UAE and MAE (among extraction methods); for PT—Pirot and Pančevo, and M method and MAE; and for PO—Rujevica and Božurna, and MAE and M methods. 

*Experimental design*. The influence of two factors (locality and extraction techniques) with two selected levels on the polyphenol yield was observed through the effects and corresponding regression coefficients of parameters and parameter interactions, as presented in Table 3. 

In the PT samples, the extraction technique (variable number 2, UAE and MAE) and interaction among locality and extraction technique have a significant effect on the TPC, while locality (variable number 1, Gulenovci and Deliblato sand) did not show statistically significant influence (Table 3). Among all the variables mentioned, the extraction technique had the most significant impact on the dependent variable (TPC). In the PP samples, both variables (locality—Pirot and Pančevo, and extraction technique—MAE and M methods) possessed a significant impact on the polyphenol yield, whereas interaction did not have a significant effect (Table 3). The extraction method had the most significant effect on the polyphenol yield, followed by locality. On the other hand, in PO, both variables (locality–Božurna and Rujevica and extraction technique–MAE and M) and their interaction showed a significant influence on the TPC, and significance followed the trend: locality > extraction technique > interaction (Table 3). The significant influence of the interaction between parameters indicated that the effect of one parameter was not the same at all levels of another parameter. Specifically, the influence of locality differed when different extraction techniques were employed. Hence, every herbal material requires the examination of the appropriate extraction procedures that will provide the highest polyphenol content in the samples. Measured and predicted values for the TPC (as the dependent variable) of the selected extracts are presented in Table S1.

According to the results of the experimental design (the measured and predicted values of the polyphenol yield in root extracts listed in Table S1), it can be seen that the highest measured polyphenol concentration was achieved under the following factors: PP (species), Pirot (locality), and MAE (extraction method), and it was 161.2 ± 2.2 mg GAE/g. The model has predicted the maximal polyphenol yield under the same parameters, PP (species), Pirot (locality), and MAE (extraction method), to be 161.1 ± 3.5 mg GAE/g. Among PT extracts, the highest TPC was reached under the following factors: Deliblato sand (locality) and UAE (extraction method), and it was 99.6 ± 0.2 mg GAE/g. The model has predicted the highest TPC under the same factors, Deliblato sand (locality) and UAE (extraction method), to be 99.6 ± 1.9 mg GAE/g. Among PO samples, the highest polyphenol yield was obtained using the following factors: Božurna (locality) and MAE (extraction method), and it was 101.2 ± 2.8 mg GAE/g. The model has predicted the highest TPC under the same factors, Božurna (locality) and MAE (extraction method), to be 101.1 ± 1.8 mg GAE/g. Since the differences between predicted and observed values (Table S1) were all less than 1%, a full factorial design may be recommended as an appropriate model for optimizing polyphenol extraction from *Paeonia* roots.

### 2.4. Total Flavonoid Content (TFC) of Root Extracts

TFC values of all prepared *Paeonia* root extracts were also determined, and the results are displayed in Table 4. Following the PT extracts, the roots from Deliblato sand were found to have the highest TFC after UAE, followed by the extracts prepared using Gulenovci roots and UAE and MAE methods and Bogovo gumno and M method (Table 4). The lowest TFC of PT extracts was detected in the samples obtained using Deliblato sand and MAE, and Bogovo gumno and the M method. TFC values in all PT extracts ranged from 0.94 to 3.27 mg catechin equivalents (CE)/g. In the case of PP extracts, the highest flavonoid yield was measured in the sample prepared using roots from Pirot and MAE and from Pančevo and UAE, followed by Južni Kučaj and MAE, while the lowest flavonoid yield was detected in the extracts prepared using roots from Bogovo gumno and UAE (Table 4). The flavonoid yield of all PP samples varied from 0.74 to 5.75 mg CE/g. The root from Rujevica was found to have the highest flavonoid concentration after the M method, followed by Božurna and the M method. The lowest TFC was measured in the samples obtained by Rujevica root and MAE or UAE (Table 4). The TFC of PO extracts was from 1.01 to 5.92 mg CE/g. The explanation for why the flavonoid yield did not follow the trend of polyphenol concentration at all examined levels can be found in the fact that the chemical composition of the herbal matrix is greatly influenced by the harvested region [16]. Additionally, it can be seen that the flavonoid yield was significantly lower in comparison to the polyphenol content in all prepared *Paeonia* root extracts, which is in agreement with the literature data. Namely, Sut et al.’s [19] study has shown that there were significant differences between the TPC and TFC of root extracts in comparison to the aerial parts of the plant. 

### 2.5. Antioxidant Activity of Root Extracts

The antioxidant capacity of all prepared *Paeonia* root extracts was examined employing two antioxidant assays: ABTS (2,2′-azino-bis(3-ethylbenzothiazoline-6-sulphonic acid) and DPPH (2,2-diphenyl-1-picrylhydrazyl radicals). The obtained results are displayed in Table 5. 

As can be seen in Table 5, in PT samples, the highest ABTS radical scavenging potential was determined in the extracts prepared from Gulenovci roots and using the M method, followed by Gulenovci and UAE, while the lowest values were obtained for the samples prepared using Deliblato sand root and MAE and Bogovo gumno root and UAE. The highest anti-ABTS activity was measured in the PT extracts obtained from Pančevo roots and MAE, followed by Bogovo gumno and the M method. On the other hand, low values were detected in the samples prepared using Golina roots and MAE (Table 5). In the case of PO, there were no differences between the extracts prepared using Božurna root and UAE, and Rujevica root and M method; thus, the mentioned extracts showed the highest ABTS radical scavenging potential. Among PO extracts, Božurna and MAE, as well as Rujevica and UAE, possessed the lowest anti-ABTS capacity (Table 5). The ABTS radical scavenging capacity did not follow the trend of polyphenol and flavonoid yields at all tested levels, which can be explained by the fact that numerous secondary metabolites (apart from polyphenols) and their interactions can have an important role in the radical scavenging potential of the plant extracts [51].

Regarding the results of the DPPH radical scavenging capacity of the root extracts, there was no statistically significant difference between the PT samples obtained using the roots from different localities and various extraction procedures (0.168–0.207 mg/mL), except in the case of Bogovo gumno (MAE), which possessed a higher value of IC_50_ (0.250 mg/mL), implying a lower anti-DPPH potential. Further, the IC_50_ values of PP and PO extracts varied in the range of 0.165 to 0.221 mg/mL and from 0.145 to 0.213 mg/mL, respectively. Additionally, there was no statistically significant difference between the anti-DPPH activity of all PP extracts, as in the case of PO extracts (Table 5). The absence of a correlation between anti-ABTS and the anti-DPPH activities of the extracts can be attributed to in the fact that the characteristics and reactivity of free radicals, as well as the mechanism of the reactions, are different. Specifically, ABTS radicals react via electron transfer, and they are more reactive than DPPH radicals, which are involved in the transfer of hydrogen atoms [53]. Hence, higher reactivity of ABTS radicals resulted in a statistically significant difference between the extracts that could not be detected using lower reactive DPPH radicals. Thus, it is important to employ various antioxidant assays to gain a better insight into the overall antioxidant potential of *Paeonia* root extracts. Since the literature data also showed a correlation between the anti-DPPH activity of the extracts and flavonoid yield [55], it can also explain the absence of statistically significant differences among various *Paeonia* root extracts in terms of DPPH radical scavenging capacity (Table 5) due to very low TFC (Table 4). 

### 2.6. Enzyme Inhibitory Activity of Root Extracts

AD, followed by diabetes mellitus 2 (DM 2), is a major global health problem in the 21st century. Today, the prevalence of AD is in progression and is estimated to sharply increase over the next 20 years [56].

Cholinesterases are the key enzymes in the synaptic cleft of the brain that terminate the cholinergic signal transfer and are considered key targets for the treatment of AD [56]. Normal cholinergic activity depends on the fast hydrolysis of the neurotransmitter acetylcholine (ACh) by cholinesterases. The fact that naturally occurring active molecules from medicinal and aromatic plants are considered to be new therapeutics has led to the discovery of secondary plant metabolites, extracts, and essential oils with the ability to inhibit AChE activity, which can cause a parallel increase in the level of ACh in the brain, reducing the symptoms of AD and PD. Based on their widespread use as neuroprotectants in conventional therapy, some publications suggest that *Paeonia* spp. could be used as potential cholinesterase inhibitors [17,19]. Therefore, this study evaluates different *Paeonia* root extracts as possible AChE and BuChE inhibitors. As can be seen in Table 6, for the AChE and BuChE assays, the highest anti-enzymatic activity was observed for the extracts of PT (obtained by MAE) collected from Bogovo gumno. These outcomes are similar to the antioxidant activity, indicating that the Bogovo gumno PT extract prepared using the M technique has a high potential to react with ABTS radicals and eliminate them. Similarly, the most effective AChE/BuChE inhibitor among the extracts of PP was one collected from Pirot (obtained by the M method). Its antioxidant activity against DPPH and ABTS radicals was assessed as moderate. The third group of extracts (PO) displayed moderate cholinesterase inhibitory potential when the UAE was implemented. Earlier studies on this topic have shown the positive effects of the polyphenols on inhibiting AChE activity [57]. Epicatechin, a monomeric flavanol, has been found to reduce tremors and enhance learning and memory ability in mouse models of PD [58]. Also, there is a claim that terpenoid-derived chemicals and other related compounds are the major responsible phytoconstituents for AChE inhibitory potential [59]. Besides, the paper of Ji and Zhang [56] suggests that flavonoids, as inhibitors of AChE and BuChE, might be functional in the treatment of AD. The extract with the highest inhibitory potential against AChE/BuChE was PT collected from Bogovo gumno (MAE), with values of 2.43 and 2.94 mg GALAE (galanthamine equivalents)/g, respectively, so it might be considered to have therapeutic potential. 

DM 2 is a metabolic disease characterized by hyperglycemia, followed by a lack of insulin, which is considered to be one of the five leading causes of death worldwide. Also, the prevalence of DM 2 has risen dramatically on the global level, which will affect 438 million people by the end of the third decade of the 21st century [60]. HPA and HIG are the key enzymes involved in the metabolism of carbohydrates and are of crucial importance for decreasing post-prandial blood glucose levels, resulting in the normal level of insulin [59]. Recent publications on this topic have shown that polyphenolic compounds and polyphenol-rich products are beneficial for preventing DM 2 [20,61]. The benefits of polyphenolic derivatives for DM 2 can be mirrored in the protection of β−cells against the toxicity of glucose; enzyme inhibitory activity through the mechanism of the action of HPA and HIG, and thus a decrease in the digestion of starch; and inhibition of the formation and accumulation of advanced glycation end products (AGEs) [62]. On the other hand, the scientific society is still in search of effective drugs from plant resources with high absorption in the GIT, a long duration of action, low toxicity, and immunostimulant effects. Therefore, the aim of this experiment is to investigate whether PT, PP, and PO contain active substances that enhance anti-diabetic activity through the inhibition of HPA/HIG (Table 6). In HPA inhibitory activity, the extract of PP (Bogovo gumno) obtained by UAE showed the highest inhibitory activity with a value of 0.26 mmol ACAE (acarbose equivalents)/g, respectively. A similar pattern was observed for PP collected from Južni Kučaj—the most effective HPA inhibitor was the extract of PP (obtained by the M method), with a value of 0.25 mmol ACAE/g. These experimental results are supported by the findings of Sut et al. [19] who reported that isoprenoid derivatives and polyphenolic compounds present in the root extract of *Paeonia arietina* and *Paeonia kesrounansis* exhibited antidiabetic activities. In addition, it was documented that gallic acid (also present in the tested *Paeonia* extracts) can reduce HPA activity, increase glucose-induced insulin secretion and glucose uptake in peripheral blood glucose uptake *in vitro*, and increase serum insulin levels and glycogen in the liver *in vivo* [61]. On the other hand, in HIG inhibitory activity, the most prominent inhibitory potential was observed in the extract of PO (Rujevica) obtained by the M method. Considering the results by the study of Sut et al. [19], it can be seen that the ethyl acetate extracts of *P. arietina* and *P. kesrounansis* possess about 40 times higher HIG inhibitory activity compared to our outputs. The potential explanation could be that some of the polyphenol compounds that cause the enzyme inhibitory potential are more soluble in ethyl acetate than in their alcoholic counterparts (in this research, ethyl alcohol (50%, *v*/*v*) was used as a solvent). Zengin et al. [63] also reported that there is a direct correlation between the nature/polarity of the solvent (due to its ability to extract target compounds) and enzyme inhibitory activity. The nature of extract (herbaceous or tree peony) and its chemical profile (ratio and concentration between secondary plant metabolites) can affect the results of enzyme-inhibitory activity as well. According to the literature review, this is the first report concerning the enzyme-inhibitory activity of extracts of PT, PP, and PO wild-grown in Serbia. Keeping this in mind, further chemical and biological studies are needed for the identification of responsible active compounds and the evaluation of the molecular mechanism of their anti-enzymatic action. 

Melanin, a major pigment in the skin, is known to protect the mentioned organ against the harmful effects of ultraviolet irradiation, oxidative stress, and DNA damage [64]. The accumulation of melanin can affect health problems associated with hyperpigmentation and other related skin disorders, such as acanthosis nigricans, melasma, and skin cancer [65]. Tyrosinase is a tetramer that contains four atoms of Cu^2+^ per molecule, which catalyzes the *o*-hydroxylation of monophenols to *o*-diphenols [64,65]. Its main drawback is the acceleration of melanogenesis, which can cause, as mentioned above, skin degeneration and malignancies [65]. Thus, the identification of novel tyrosinase inhibitors was crucial to the development of new therapeutic agents that may be used to treat diseases associated with skin-pathological disorders. Many active compounds, including kojic acid, vitamin C, hydroquinone, and arbutin, are reported to exhibit inhibition of tyrosinase and are extensively used in pharmacology, clinical research, and similar fields [66]. However, due to their lack of safety, unsatisfactory efficacy, and cytotoxicity, further studies for proper use are exclusively necessary.

Regarding that, the presented findings revealed that all examined extracts of PT, PP, and PO inhibited tyrosinase (Table 6). Among all tested extracts, the extract of PO (Rujevica) obtained by the M method, followed by the extracts of PP (Južni Kučaj and Pirot) obtained by UAE and the M method, displayed the highest tyrosinase inhibition. Previous investigations showed that root extracts of *P. arietina* and *P. kesrounansis* show high tyrosinase-inhibitory activity, about four times higher compared to our results [19]. Also, in the literature, it can be found that other organs of *Paeonia* species, specifically the aerial parts of *P. mascula* ssp. *hellenica,* possess a strong potential to inhibit tyrosinase [67]. The reason for this atypical occurrence could be that the synthesis of secondary plant metabolites—tyrosinase inhibitors, such as flavonoids and galloyl derivatives—in aerial parts of peony is more intense compared to the roots, which are not directly exposed to sunlight. However, further investigations are necessary to support this claim. In addition, kaempferol-3-*O*-(6-β-*O*-galloyl-β-D-glucopyranoside) isolated from the flowers of *P. lactiflora* was found to inhibit tyrosinase, with greater effectiveness compared to reference anti-tyrosinase inhibitors (e.g., kojic acid) [19,67]. 

### 2.7. Molecular Docking

**Figure 3:**
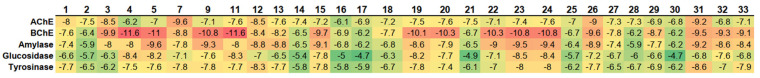
Docking scores of 33 compounds identified in the *Paeonia* root extracts to five enzymes. The red color indicates a higher binding affinity. AChE: acetylcholinesterase; BuChE: butyrylcholinesterase.

**Figure 4:**
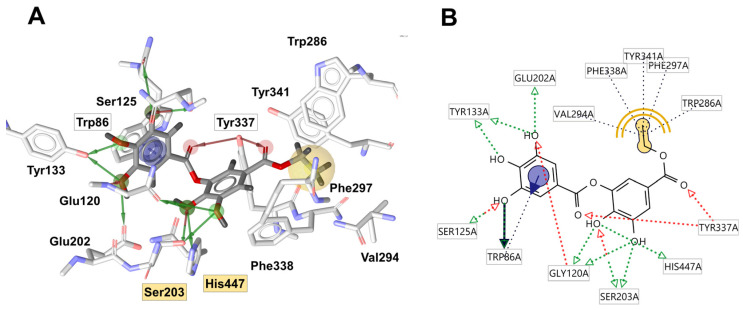
(**A**) Binding mode of ethyl digallate (**7**) within the acetylcholinesterase binding pocket; (**B**) 2D ligand interaction diagram for this macromolecular complex. Hydrogen bond donor, hydrogen bond acceptor, hydrophobic, and π–π stacking interactions are represented by green arrows, red arrows, yellow spheres, and blue rings, respectively.

**Figure 5:**
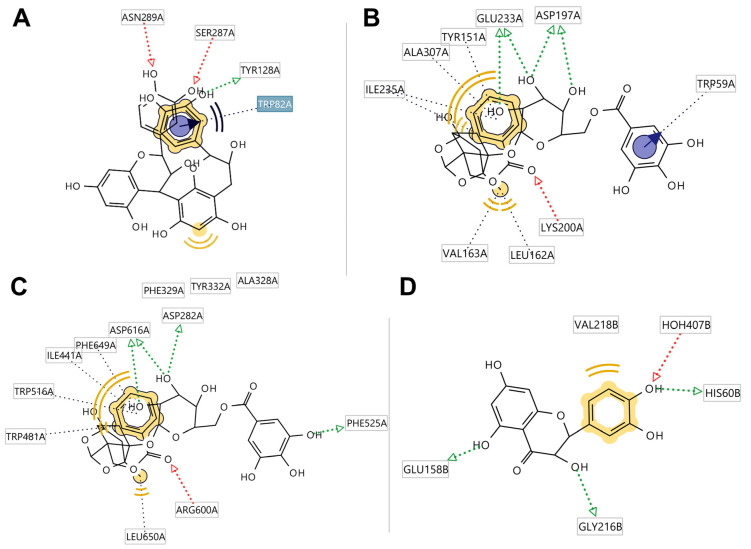
2D ligand interaction diagrams showing the most important interactions that stabilize the protein–ligand complexes depicted for: (**A**) butyrylcholinesterase-B-type procyanidin dimer 2, (**B**) amylase-galloyl-paeoniflorin, (**C**) glucosidase-galloyl-paeoniflorin, and (**D**) tyrosinase-taxifolin. Hydrogen bond donor, hydrogen bond acceptor, hydrophobic, and π–π stacking interactions are represented by green arrows, red arrows, yellow spheres, and blue rings, respectively.

The chemical profile of the *Peaonia* root extracts was analyzed by molecular docking on five enzymes (AChE, BuChE, HPA, HIG, and tyrosinase) with the aim of highlighting individual compounds that contribute most to the observed activities of the extracts. The heatmap representation of calculated binding affinities is shown in Figure 3. The binding modes of several protein–ligand complexes are presented in Figure 4 and Figure 5, indicating the most important interactions that stabilize these structures. 

Ethyl gallate (**7**) and paeoniflorigenone (**26**) exhibited notable binding affinities toward AChE (–9.6 and –9.0 kcal/mol, respectively). Ethyl gallate interacts with Ser203 and His447 from the catalytic triad by serving as a hydrogen-bond donor (HBD) (Figure 4). Additionally, it binds to Trp86 from the anionic site via HBD and π–π stacking interactions and to Tyr337 from the peripheral anionic site (PAS) through hydrogen-bond acceptor (HBA) interactions involving two carbonyl oxygens. Moreover, the ethyl group establishes a rich pattern of hydrophobic interactions with Val294, Phe338, Tyr341, Phe297, and Trp286. 

In addition, pentagalloyl-hexose (**4**) and B-type procyanidin dimer 2 (**11**) displayed the highest binding activities of –11.6 kcal/mol toward BuChE. The ligand-interaction diagram for the BChE-**4** complex (Figure 4A) illustrates interactions with Trp82 and Phe329 from the anionic site, as well as with Tyr332 from the PAS, which stabilize this structure. Galloyl-paeoniflorin (**23**) showed the highest binding affinities toward amylase and glucosidase (–9.5 and –8.5 kcal/mol, respectively) among all compounds identified in the chemical profile of root extracts, indicating the significant role of *Paeonia* terpenes in the overall biological activities. The most important protein–ligand interactions for binding to these two enzymes are displayed in Figure 4B and Figure 5C. Finally, taxifolin (**31**) exhibited the highest binding affinity to tyrosinase (–8.6 kcal/mol), attributed to hydrogen-bonding interactions of the taxifolin B-ring with His60 and catalytic water 407, which bridges two cooper cations within the active site of the enzyme. 

In order to fully explore the pharmaceutical potential of the high-affinity compounds identified through molecular docking, it is essential to conduct experimental validation of their binding affinity and binding kinetics. Furthermore, optimizing their pharmacokinetic properties to enhance drug-likeness is likely necessary for the development of viable drug candidates. 

### 2.8. Antibacterial Activity of Root Extracts

In humans, *Listeria monocytogenes*, *Staphylococcus aureus*, and *Escherichia coli* are of the GIT and urogenital tract (UGT), including gastroenteritis, hemorrhagic colitis, urinary infections, and other GIT- and UGT-related disorders [68,69]. While the use of synthetic drugs has lowered the severity and spread of many infectious diseases, food toxoinfections remain the leading cause of death in both high- and low-income countries. Synthetic anti-inflammatory medications have come under investigation due to the recent advances in our comprehension of the human GIT, digestion, intestinal peristalsis, and similar. 

The bacteria selected for the present study were chosen based on several factors. First, *L. monocytogenes* NCTC 7973, *S. aureus* ATCC 11632, and *Bacillus cereus* human isolate were selected due to their significance as common pathogens known to cause foodborne illnesses and infections in humans. Additionally, these bacteria represent a range of Gram-positive species, providing insight into the efficacy of the tested antimicrobial agents against this bacterial group. Furthermore, three Gram-negative bacteria species were included, namely, *Salmonella* Typhimurium ATCC 13311, *Pseudomonas aeruginosa* ATCC 27853, and *E. coli* ATCC 25922. These bacteria were chosen to broaden the scope of the study and assess the antimicrobial activity against Gram-negative pathogens, which are also prevalent causes of various infectious diseases. Finally, the selection of these bacterial strains aimed to provide a comprehensive evaluation of the antimicrobial agents under investigation across different bacterial types, encompassing both Gram-positive and Gram-negative species commonly associated with human infections and food contamination. Some literature reports suggest that polyphenols (phenolic acids, flavonoids, anthocyanins, condensed tannins, etc.) are the main class of active compounds in medicinal plants associated with antibacterial activity [70,71]. Also, the antibacterial activity can be attributed to the number and position of hydroxyl and carboxylic groups in the benzoic ring of phenol-carboxylic acids [16,70]. Much effort has been focused on plant medicinal formulations and their bioconstituents as potential sources of valuable antibacterial agents for the prevention or eradication of *Salmonella*, *E. coli*, and other pathogens. In particular, it was reported that some *Paeonia* species had growth-inhibitory activity toward human GIT pathogens [72]. Herein, water–ethanolic extracts of PT, PP, and PO were analyzed as a potential source of antibacterial agents intended for use in the therapeutic treatment of the disease of human GIT. 

The results of the antibacterial activity tested by the broth microdilution method are summarized in Table 7. The antimicrobial assay showed that extracts of PT, PP, and PO can inhibit the growth of the tested bacterial strains. Minimal inhibitory concentration (MIC) values range from 0.125 to 4.0 mg/mL, and minimal bactericidal concentration (MBC) values range from 0.25 to 8.0 mg/mL. The PP extract (Južni Kučaj) obtained by the M method had the highest antibacterial activity against *B. cereus* (Gram-negative), followed by roots from Golina and Bogovo gumno (both prepared by M method). Further, the PO extract (Rujevica) obtained by the M method shows a lower ability to inhibit bacterial growth, being the most effective against *L. monocytogenes* (Gram-positive) and *P. aeruginosa* (Gram-negative). Similarly, the extract of PT shows slightly poorer antimicrobial activity compared to PP and PO, except for roots originating from Bogovo gumno (obtained by UAE and M). 

The extraction method proven to produce the most effective antibacterial agents is the M method, due to the fact that the M method is a cold extraction method, without an aggressive regime of extraction, so the high ratio of preservation of polyphenols, the carriers of antimicrobial activity, is completely enabled. 

On the other hand, the results of phytochemical screening suggest that the obtained macerates of PT, PP, and PO abound in the content of phenolic acid, flavonoids, and other phenol-core-related substances. To date, the exact mode of action of polyphenols or other bioactive molecules found in the different herbaceous *Paeonia* root extracts on bacteria has not been well documented. Namely, there are several hypotheses suggesting that polyphenols from plant sources can disrupt the bacterial cell wall and cytoplasmic membrane, which leads to the dissolution of the protein fraction and the leaching out of essential ions/biomolecules (K^+^, PO_4_^3−^, nucleic acids, etc.), reducing the cellular activity of bacteria [73,74,75]. Flavonoids can inhibit cell membrane functions, energy metabolism, and the synthesis of DNA and RNA material through the mechanism of linkage with the soluble protein fractions located outside the cell walls of bacteria, or nucleic material might be broken down into small pieces after the flavonoids enter the cell [76,77]. 

Based on the results of our experiment, it is evident that the tested extracts of PP and PO exhibit potent antibacterial activity and contribute to significantly lower survival of bacteria, which are the most common mediators of health problems in human GIT. According to Aligiannis et al. [78], extracts with MIC values up to 0.5 mg/mL can be classified as strong inhibitors of bacteria and can be useful for therapeutic applications. 

Furthermore, a correlation between the achieved antibacterial activity of the tested root extracts and their chemical assembly insinuates that the observed activity can be attributed to the high presence of the major extract phytoconstituents (e.g., gallic acid, catechin, myristicin, and taxifolin). Since the extracts of PT, PP, and PO are complex and impure mixtures, their antibacterial activities vary in accordance with the biological molecules that they include. Moreover, strong antibacterial activity can be a result of the synergistic effect that is formed by the active molecules coming together. There is also an assumption that some differences in antibacterial activity observed for three *Paeonia* root samples might be attributed to environmental factors and/or possible plant infection that might interfere during the synthesis of antibacterial active compounds [74]. 

Finally, the comparison of all determined antibacterial effects indicated that the general susceptibility of the pathogens in the tested extracts of PT, PP, and PO could be given in the following order: the most sensitive were isolates of *B. cereus,* followed by *P. aeruginosa, E. coli,* and *L. monocytogenes.* As mentioned, it seems that the M method had a superior effect on the preservation of polyphenols compared to the other two extraction techniques, enabling the best impact on the antibacterial activity of root extracts. 

### 2.9. Simulated In Vitro GID and Bioaccessibility 

A total of 58 bioactive compounds (BCs) were identified in various water extracts—tea samples before and after *in vitro* gastrointestinal digestion (GID). All identified BCs were categorized into different classes and subclasses: (1) phenolic acids and derivatives (primarily gallic acid, ellagic acid, and their derivatives); (2) flavonoid and derivatives (primarily flavan-3-ols and procyanidins); and (3) terpenoids of *Paeonia* root. The relative content of total identified terpenoids was similar in all control tea samples (30.53–34.66%), mainly due to the high relative content of paeoniflorin (Table 8). On the other hand, the relative contents of total phenolic acids and flavonoid derivatives in control tea samples were different and strongly depended on the peony root species (Table 8). Thus, flavan-3-ols and procyanidins (catechin, chalcan flavan-3-ol dimer isomer I, and B-type procyanidin dimer and trimer isomers) predominated in the PTc (control (initial) PT tea) and PPc (control (initial) PP tea samples) (46.39–49.82%), while gallic acid derivatives (33.59%) and ellagic acid (4.18%) were the most abundant in POc (control (initial) PO tea) (Table 8). The PTc control tea had the highest total relative content of identified BCs, while their relative content was lower at 7.63% and 21% in the PPc and POc control tea samples, respectively.

The recovery of total and individual BCs of the tea samples after *in vitro* gastrointestinal digestion is shown in Table 9. As can be seen, a total recovery of all identified BCs was reduced and similar for all digested tea samples (DPT–digested of PT tea, DPP–digested of PP tea, and DPO–digested of PO tea) and ranged from 71.75 to 81.91%. These results are higher or similar to those reported by Qin et al. [79] and Koláčková et al. [80] for the recovery of total phenolic compounds of green tea infusion after GID (68.71% and 62–84%, respectively), but they are significantly higher than the recovery of total phenolic compounds of teas prepared from the flowers of *P. lactiflora* (21.25%) [81] and *P. suffruticosa* (12.63%) [82]. On the other hand, an increase in the total amount of total phenolic compounds was found for Guayusa after an *in vitro* GID of 3% [83]. However, the recovery for each identified compound was different and was highly dependent on their structure, their tendency to react with digestive enzymes, solubilization in the digestive cocktail, and their presence in the initial sample [84,85,86]. The majority of the identified phenolic acid derivatives showed reduced recovery, with the exception of methyl gallate and methyl digallate, whose relative content (Table 8) and recovery (Table 9) increased significantly after *in vitro* GID. It is known that peony root is a rich source of various galloylated macromolecules (tannins or some carbohydrates) from which the methyl gallate derivatives were probably released. This is also confirmed by the significantly lower recovery of all identified high-molecular-weight galloyl derivatives (compounds **8**–**16**, Table 9). In addition, some high-molecular-weight galloyl derivatives were digested but not detected in the initial tea samples, as they were probably removed by filtration prior to chromatographic analysis. The high percentage of released methyl gallate in the digested tea samples was due to the high recovery of total phenolic acid derivatives (56.53–86.30%). A high recovery of phenolic acid derivatives after GID was also observed by Kelebek et al. [83] for Guayusa tea. These results may contribute significantly to the future evaluation of the biological activity of peony root tea, as methyl gallate has been shown to have a variety of biological functions [87]. Ellagic acid and its derivatives exhibited lower recovery after *in vitro* GID (Table 9), except for the recovery of dimethyl and trimethyl ellagic acid (DPP and DPO). However, their relative contents in the initial PPc and POc were low (about 1%, Table 8), so they have no significant effect on the overall recovery of total phenolic acid derivatives. As expected, the flavan-3-ols and procyanidins showed lower recovery, as these molecules have a high tendency to react with enzymes and compounds of the digestive cocktail [86,88]. Other authors also reported a low recovery of flavan-3-ols from green tea flowers [80,89] and *P. lactiflora* Pall. flower tea [81]. Other detected flavonoids were present in trace amounts, so their recovery has a negligible effect on the recovery of total flavonoid derivatives. The majority of the detected monoterpenoids (compounds **44**–**49**, Table 9) and galloyl/benzoyl paeoniflorin derivatives (compounds **55**, **56**, and **58**, Table 9), showed low recovery or were not found in digested tea samples. However, the predominantly found paeoniflorin and its derivatives (oxypaeoniflorin, paeoniflorigenone, with the exception of DPP, and the selectively detected mudanpioside) showed a reduced recovery after *in vitro* GID. Albiflorin, on the other hand, showed an increased recovery, as it was probably released during digestion.

It is worth mentioning that in vivo studies have reported low bioavailability of paeoniflorin, only 2.3% [90], which is due to poor permeation and its hydrolysis by the gut mircrobiota to various metabolites such as benzoic acid, paeoniflorgenin, 7S- or 7R-paeonimetabolin I, paeonimetabolin II, and sinomenine [91]. 

## 3. Materials and Methods

### 3.1. Origin of Plant Material

The fresh roots of PT, PP, and PO were collected from eleven localities in Serbia. PT were collected from Gulenovci, Deliblato sands, and Bogovo gumno, PP from Pirot, Južni Kučaj, Golina, Bogovo gumno, Krivi vir, and Pančevo while PO were from Rujevica and Božurna. The roots were collected at the end of the vegetation, in September 2022. As all peonies in the Republic of Serbia are protected by law, their collection was conducted with appropriate permission (353–01-162/2022–04, issued on 24 February, Ministry of Environmental Protection of the Republic of Serbia). The voucher specimens of these medicinal plant species were deposited in the herbarium BUNS of the Department of Biology and Ecology at the University of Novi Sad, Faculty of Sciences (Novi Sad, Serbia), where the identification confirmation was performed. Prior to extraction, the collected roots were left to dry in a shaded room for 30 days at 20 °C. Dried roots were ground in the mill (M-20, IKA Universal mill, IKA^®^, GmbH&Co., Königswinter, Germany) and separated by a sieve to obtain fine particles with a size of 0.75 mm. The powdered roots were stored in a refrigerator (4 °C).

### 3.2. Chemicals and Reagents

Ethyl alcohol (Zorka Pharma, Šabac, Serbia, 96%, *v*/*v*) and distilled water, as a mixture, were used as extraction agents. Methyl alcohol (HPLC grade), Folin-Ciocalteu reagent, gallic acid, catechin hydrate, aluminum chloride, hydrochloric acid, sodium nitrate, sodium chloride, sodium hydroxide, sodium hydrogen carbonate, 6-hydroxy-2,5,7,8-tetramethylchroman-2-carboxylic acid (Trolox), potassium persulfate, salivary α-amylase solution, pancreatin from porcine pancreas, bile acid mixture from bovine and ovine mucin, mucin, and pepsin from porcine stomach were purchased from Sigma-Aldrich, Darmstadt, Germany. L-3,4-dihydroxyphenylalanine, 5,5′-Dithiobis(2-nitrobenzoic acid), acetylcholinesterase from *Electrophorus electricus* (electric eel) Type-VI-S (EC 3.1.1.7), butyrylcholinesterase from equine serum (EC 3.1.1.8), acetylthiocholine iodide, butyrylthiocholine chloride, kojic acid, alpha-amylase *ex-porcine* pancreas (EC 3.2.1.1), acarbose, *p*-iodonitrotetrazoliumviolet (>95%), α-glucosidase solution from *Saccharomyces cerevisiae* (EC 3.2.1.20), Lugol reagents (diluted iodine-potassium iodide solution), ABTS, DPPH, tryptic soy broth (TSB), and formic acid were obtained by Sigma Aldrich, St. Louis, MO, USA. Gentamicin (solution for injection, 40 mg/mL) was bought from Panfarma (Belgrade, Serbia).

### 3.3. Extraction

#### 3.3.1. MAE

MAE was performed using a method previously reported by Batinić et al. [17]. MAE was carried out at 100 °C using a microwave reactor (Milestone ETHOS X, 2.45 GHz, Milestone, Italy) equipped with a digital infrared temperature sensor that monitors the process temperature and two magnetrons with a maximum operative power of 1.8 kW. All experiments were conducted in three cycles, in the SR-15 rotor with a power of 0.7 kW, in a closed poly(tetrafluoroethylene) Teflon vessel (0.1 L), using a magnetic stirring bar (12 × 30 mm) for uniform mixing (the mixing speed was software-controlled; magnetic stirring of solution up to a speed of 3400 rpm). Ethyl alcohol with 50% water, an extraction time of 2 min, and a solid-to-solvent ratio of 1:10 were employed in the MAE. Following the extraction process, permeate was separated from retentate using a laboratory glass funnel and cellulose-acetate filter (25 mm, 0.45 µm). The solvent was removed using vacuum evaporation (Rotavapor Heidolph 4001-efficient, Heidolph Instruments, Schwabach, Germany) at a temperature of 45 °C and a pressure of 0.180 ± 0.05 bar. The extracts were stored in the refrigerator at 4 °C before analysis.

#### 3.3.2. UAE

UAE was performed in the ultrasound bath (Digital ultrasonic cleaner bath, DU-32, 40 kHz, 100 W, Argo LAB, Carpi—MO, Italy) with a maximal useful volume of 3.2 L. The method was previously described by Batinić et al. [17]. The roots were placed in a 0.1 L flask, and an extraction agent, ethyl alcohol (50%, *v*/*v*), was added (the solid-to-solvent ratio was 1:10). The Erlenmeyer flask with a sample was immersed in the ultrasonic bath and sonicated for 30 min at 30 °C. After sonication, the extracts were filtered through the cellulose-acetate filter (25 mm, 0.45 μm), while the solvent was removed using a rotary vapor evaporator at 45 °C at 0.180 ± 0.05 bar. The extracts were stored at 4 °C prior to analysis. 

#### 3.3.3. M Method

The M method in an Erlenmeyer flask (0.1 L) was performed on a tube roller mixer (Stuart, SRT-6, Germany) with agitation fixed at 200 rpm, at 30 °C [17]. The volume of extraction agent (ethyl alcohol, 50%, *v*/*v*) was 0.01 L, the solid-to-solvent ratio was 1:10, and the extraction time was 30 min. Dry extracts were obtained using vacuum evaporation, as described in the previous section. The extracts were stored at 4 °C until further measurements.

#### 3.3.4. Preparation of Hot Water Extracts (Teas)

PT (Gulenovci), PP (Pirot), and PO (Rujevica) were selected for the study of *in vitro* simulated GID as they proved to be most effective against tested pathogens, and they also have significant enzyme-inhibitory potential. Also, as previous tests show, they possess a prominent potential to neutralize ABTS and DPPH radicals. Finely ground root samples (4 g) were mixed with 40 mL of boiling water and gently stirred on a mechanical shaker for 30 min. The hot water extracts were prepared according to the standard procedure for the preparation of teas from the roots of *Althaea officinalis* [92]. The samples were then centrifuged at 4000 rpm for 5 min, and liquid fractions (teas) were freeze-dried and used for *in vitro* gastrointestinal digestion. 

### 3.4. Chemical and Structural Analysis

#### 3.4.1. UHPLC-LTQ-OrbiTrap MS Analysis

Selected *Paeonia* extracts obtained using the UAE method are included in the UHPLC-LTQ-OrbiTrap MS analysis with the aim of identifying and quantifying their individual compounds. The experiments were performed using a liquid chromatography system (Thermo Fisher Scientific, Karlsruhe, Germany), which consisted of an Accela autosampler and quaternary Accela 600 pump connected to the orbitrap hybrid mass spectrometer (LTQ OrbiTrap XL, Thermo Fisher Scientific, Karlsruhe, Germany) with heated electrospray ionization, operating in positive and negative ionization modes. Separations were conducted on a Syncronis^TM^ C18 reversed-phase column (50 × 2.1 mm, 1.7 µm) from Thermo Fisher Scientific (Karlsruhe, Germany). The molecular editor software ChemDraw (ChemDraw 12.0, Waltham, MA, USA) was used for drawing the structure of organic molecules and for calculating the molecular masses of the active compounds of interest. The software Xcalibur (Xcalibur 2.1, Thermo Fisher Scientific, Waltham, MA, USA) was used for instrument control, data acquisition, and data analysis. Deprotonated molecule mass [M-H]^−^ and MS^2^, MS^3^, and MS^4^ fragmentation patterns were used for the identification of active compounds in the extract with the assistance of the available spectroscopic, accurate mass, and MS fragmentation data from the literature [15,16,19,20,93]. 

#### 3.4.2. FTIR Analysis

FTIR spectroscopy is an effective and non-destructive method of instrumental analysis for the differentiation and characterization of organic or inorganic materials, natural products, drugs, etc. by observing the individual bands or functional groups to precisely identify the molecular conformations, bonding types, and physical or chemical interactions that compose the sample. FTIR spectral analysis was recorded using an ATR-IR spectrometer (Nicolet^TM^ iS^TM^, Thermo Fisher Scientific, Waltham, MA, USA) in the wavenumber range between 500 and 4000 cm^−1^, with 64 accumulations, and a resolution of 4 cm^−1^, at 25 ± 5 °C. The method was previously described by Batinić et al. [17]. The FTIR analysis was performed by fixing 20 mg of dry root extract (prepared by vacuum evaporation as previously described) to a brazed diamond crystal plate for smart orbit (Thermo Fischer Scientific, Waltham, MA, USA). FTIR spectra were recorded in ATR mode and corrected by the OMNIC spectroscopy software (OMNIC™ Series software 9.8.635, Thermo Fisher Scientific, Waltham, MA, USA). A graphic view of spectra and deconvolution were conducted in the OriginPro 9.0 software (OriginLab Co., Northampton, MA, USA).

#### 3.4.3. Determination of TPC

The TPC in the dry root extracts of PT, PP, and PO was determined by a modified Folin-Ciocalteu method [16,94]. The extract (0.02 mL, 1 mg/mL) was mixed with 0.1 mL of Folin-Ciocalteu phenol reagent (previously diluted 2-fold with distilled water), after which 0.3 mL of sodium carbonate solution (20%, *w*/*v*) was added, and the mixture was made up to 2 mL with distilled water. After 120 min of incubation at 25 ± 5 °C, the absorbance (λ_max_ = 765 nm) was read against a blank using a UV/Visible scanning spectrophotometer (UV/Vis 1800, Shimadzu, Tokyo, Japan). The analytical standard of gallic acid (GA) was used for the construction of the calibration curve (0.1–1.6 mg/mL), while the amount of polyphenols was expressed as milligrams of GA equivalents per gram of dry extract (mg GAE/g).

#### 3.4.4. Determination of TFC

The TFC in the dry root extracts of PT, PP, and PO was estimated colorimetrically using the aluminum chloride colorimetric assay based on the experimental procedure given by Shraim et al. [95]. In brief, an aliquot of 0.25 mL of extract (1 mg/mL) was mixed with 0.075 mL of sodium nitrite solution (5%, *w*/*v*) and 1.25 mL of distilled water. After 5 min, 0.15 mL of aluminum chloride solution (10%, *w*/*v*) was added to the mixture, and 1 min later, 0.5 mL of sodium hydroxide solution (1 mol/L) was also added to complete the reaction. The final volume was made up of 3 mL of distilled water. Then, the mixture was gently mixed (Vortex 3, IKA^®^, Königswinter, Germany) and the tube containing the mixture was kept in the dark for 30 min prior to UV/Vis analysis. The analytical standard of catechin (CA) was used for the construction of the calibration curve (0.037–0.3 mg/mL). The absorbance (λ_max_ = 510 nm) was measured against a blank using a UV/visible scanning spectrophotometer, while TFC was expressed as CA equivalents per gram of dry extract (mg CAE/g).

### 3.5. Determination of Free Radical Scavenging Activity

#### ABTS and DPPH Assays

The free radical scavenging activity of the liquid root extracts (obtained at different extraction methods) was determined using two assays—ABTS and DPPH tests. The basis of the ABTS assay is the interaction between polyphenol antioxidants and the pregenerated ABTS^•+^ radical cation. ABTS^•+^ can be easily quantitatively detected due to the bleaching of absorption spectrum characteristic maxima at 734–757 nm, depending on the solvatochromic effects of the solvent used for extraction. Usually, ABTS^•+^ is pregenerated a day before by mixing ABTS^•+^ solution (4 × 10^−3^ g/mL) with 0.088 mL of potassium persulfate (2.45 × 10^−3^ mol/L) and standing overnight (12–16 h) at 0–4 °C to complete the reaction of radical activation (the end of the reaction can be easily observed by a color change from deep to bluish-green). Following activation, the ABTS stock solution was diluted with ethyl alcohol (50%, *v*/*v*) to reach an absorbance of 0.700 ± 0.020 at 753 nm. Then, 2.8 mL of ABTS stock solution was mixed with 0.2 mL of extract, while the blank was prepared by mixing the same amount of ABTS stock solution with 0.2 mL of extraction agent (ethyl alcohol, 50% *v*/*v*). After 30 min of incubation in the dark at 20 ± 5 °C, the antioxidant activity of the extracts was determined by measuring the absorbance compared to the blank. The radical scavenging activity (RSA_ABTS_) was calculated according to the following equation:

(1) $${\mathrm{RSA}}_{\mathrm{ABTS}}\;(\%)=\;\frac{A_{0}-Ax}{A_{0}}\times 100$$

where A_0_ represents the absorbance of ABTS^•+^ solution, while A_x_ is the absorbance of ABTS^•+^ solution and the extract. Trolox was used as a standard for the calibration curve (0.030–1.0 mg/mL). The RSA_ABTS_ was expressed as µg Trolox equivalents (TE) per mL of extract (µg TE/mL).

The principle of the DPPH assay is based on the reduction of DPPH^•^, which has an absorption maximum at 517 nm. The DPPH^•^ stock solution was prepared by dissolving 0.252 mg of DPPH in 9 mL of ethyl alcohol (96%, *v*/*v*). For the photometric assay, 2.8 mL of DPPH^•^ stock solution and 0.2 mL of extract were mixed. The blank was prepared by mixing 2.8 mL of DPPH^•^ stock solution with 0.2 mL of ethyl alcohol (50% *v*/*v*). The absorbance reading was taken after 30 min of incubation in a dark place (20 ± 5 °C) against a blank at 517 nm. The radical scavenging activity (RSA_DPPH_) was calculated according to the following equation:

(2) $${\mathrm{RSA}}_{\mathrm{DPPH}}\;(\%)=\;\frac{A_{0}-Ax}{A_{0}}\times 100$$

where A_0_ represents the absorbance of DPPH^•^ solution and extraction solvent, while A_x_ is the absorbance of DPPH^•^ solution and the extract. The results were expressed as the concentration of extract required to neutralize 50% of DPPH^•^ (IC_50_, mg/mL).

### 3.6. Determination of Enzyme Inhibitory Activities

#### 3.6.1. AChE and BuChE Enzymatic Assays

According to Grochowski et al. [96], the reaction mixture composed of 0.05 mL of root extract (1 mg/mL), 0.125 mL of 5,5′-dithiobis(2-nitrobenzoic acid) (3 × 10^−3^ mol/L), and 0.025 mL of AChE (0.265 U/mL), or BuChE (0.026 U/mL) Tris-hydrochloride buffer solution (pH 8.0) was added to the substrates (ACh, 0.015 mol/L or butyrylthiocholine, 1.5 × 10^−3^ mol/L). Moreover, a blank (prepared in the same manner but without the root extract) was prepared, and all absorbances were read at 405 nm after 15 min. The standard inhibitor for cholinesterases was galantamine, and milligrams of galantamine equivalents per gram of extract (mg GALAE/g) was the measurement unit. 

#### 3.6.2. HPA Enzymatic Assay

According to Sut et al. [19], after 10 min of incubation at 37 °C, the reaction mixture comprising the root extract (0.05 mL, 1 mg/mL) and HPA solution *ex-porcine* pancreas (0.05 mL, 10 U/mL) in phosphate buffer (pH 6.9, with 6 × 10^−3^ mol/L of sodium chloride) was added to a starch solution (0.05 mL, 0.05% *w*/*v*). A blank was made by adding the test solution to all reagents without root extract. The reaction was ended with the addition of 0.025 mL of hydrochloric acid (1 mol/L) and 0.1 mL of iodine-potassium iodide (Lugol’s reagent). After 10 min of incubation at 37 °C, all of the absorbances were recorded at 630 nm. The results were expressed as millimoles of acarbose equivalents per gram of extract (ACAE/g).

#### 3.6.3. HIG Enzymatic Assay

The HIG enzymatic assay was performed following the experimental procedure given by Grochowski et al. [96]. In brief, 0.05 mL of the root extract (1 mg/mL) was mixed with 0.05 mL of glutathione (0.5 mg/mL), 0.05 mL of HIG (0.2 U/mL) in phosphate buffer (pH 6.8, 1 mol/L), and 0.05 mL of 4-N-trophenyl-α-D-glucopyranoside (0.1 mol/L). The reaction was stopped with the addition of 0.05 mL of sodium carbonate (0.2 mol/L). Moreover, the blank was prepared in the same manner but without root extract. The absorbances were recorded at 400 nm (with a 96-well microplate reader, Agilent Technologies Epoch, Winooski, VT, USA) after 15 min of incubation at 37 °C. Millimoles of acarbose equivalents per gram of extract (mmol ACAE/g) were the measurement unit. 

#### 3.6.4. Tyrosinase Enzymatic Assay

The tyrosinase enzymatic assay was carried out following the instructions provided by Grochowski et al. [96]. Specifically, 0.025 mL of extract solution of root extract (1 mg/mL) was mixed with 0.04 mL of tyrosinase (200 U/mL) and 0.1 mL of phosphate buffer (pH 6.8, 0.04 mol/L) in a 96-well microplate and incubated for 15 min at 25 °C. Then, the reaction was initiated using L-3,4-dixydroxyphenylalanine (0.04 mL, 0.01 mol/L), and after 10 min of incubation at 25 °C, the absorbance was read at 492 nm. A control was prepared in the same manner, but without tyrosinase. The tyrosinase enzymatic assay was determined by subtracting the absorbance of the control from that of the test solution, and the results were expressed as milligrams of kojic acid equivalents per gram of extract (mg KAE/g).

### 3.7. Molecular Docking

The initial structures of macromolecular targets in complexes with corresponding inhibitors were downloaded from the Protein Databank (PDB). Specifically, the following PDB structures were utilized: 4 TPK for AChE [97], BuChE [98], 4EY5 for 1OSE for HPA [99], 5NN8 for HIG [100], and 3NQ1 for tyrosinase [101]. The ionization states of the enzymes were adjusted to pH 7.40 using PROPKA [102]. Prior to simulations, we removed all water molecules and co-crystallized small molecules, creating space for the binding of the studied compounds. Vega ZZ 3.2.3. was used as the GUI [103]. The 2D structures of all compounds identified in the root extracts of three *Paeonia* species were either downloaded from PubChem or sketched in ChemDraw. Initial conformations of these compounds were generated using the MMFF94s force field in LigandScout 4.4 [104]. Subsequently, the final structures were optimized using the semiempirical PM7 method [105] implemented in MOPAC2016 [106], incorporating the COSMO solvation model of water and applying the PRECISE option to increase the accuracy. The final structures of all ligands were stored in the .sdf database. To identify the active site of each protein for molecular docking, we selected all residues within 10 Å from the co-crystallized inhibitor. AutoDock Vina 1.1 was chosen for the molecular docking process [107]. The virtual screening module within Vega ZZ 3.2.3 was used to study the binding affinities of the natural product database against each of the five enzymes. The exhaustiveness was set to 25, and 5 binding modes were saved for each ligand.

### 3.8. Determination of Antibacterial Activity

#### 3.8.1. Selection of Microorganisms and Culture Conditions

Antibacterial activity was tested against six bacterial strains from the National Collection of Type Cultures (NCTC) and the American Type Culture Collection (ATCC). For the bioassays, three Gram-positive bacteria species, namely, *L. monocytogenes* NCTC 7973, *S. aureus* ATCC 11632, and *B. cereus* human isolate, and three Gram-negative bacteria species, namely, *S.* Typhimurium ATCC 13311, *P. aeruginosa* ATCC 27853, and *E. coli* ATCC 25922, were used. All tested bacteria were from the Laboratory of Mycology, Department of Plant Physiology, University of Belgrade, Institute for Biological Research “Siniša Stanković”, Belgrade, Serbia. Bacteria were maintained on Mueller–Hinton agar and stored at 4 °C.

#### 3.8.2. Broth Microdilution Method

In order to investigate the antibacterial activity of the root extracts, the modified broth microdilution method was used [15,16]. Bacterial species were cultured for 24 h at 37 °C in a Tryptic soy broth (TSB) medium. The fresh bacterial suspension was adjusted with sterile saline to a concentration of 1.0 × 10^8^ per well. All root extracts were dissolved in ethyl alcohol (30%, *v*/*v*) to prepare a root extract (CFU) colony-forming unit stock solution with a concentration of 10 mg/mL. Minimal inhibitory concentration (MIC) and minimal bactericidal concentration (MBC) determinations were conducted by a serial dilution method using 96-well microtiter polystyrene plates. The MIC and MBC were conducted by serial subcultivation of 0.16 mL of extract into microtiter plates containing 0.04 mL of broth per well, followed by the addition of 0.01 mL of bacterial suspension and further incubation for 24 h at 37 °C; the MIC/MBC values were obtained following the addition of 0.02 mL of *p*-iodonitrotetrazolium violet (0.2 mg/mL) and incubation at 37 °C for 30 min. The lowest concentrations without visible growth were taken as MIC values, while the MBC indicates the lowest concentrations of root extracts at which the original inoculum was killed by 99.5%. A positive control, the antibiotic Gentamicin^®^, was used in the experiment. 

### 3.9. Simulated In Vitro GID and Bioaccessibility

Prepared teas of selected *Peaonie* root samples (PO, PP, and PT) were subjected to a static *in vitro* GID with the aim of determining the recovery of specific bioactive compounds. The lyophilized powders from each infusion were completely dissolved in 5 mL of milli-Q water (dissolved teas) and further used for control and *in vitro* GID. *In vitro* GID was performed following the protocol previously reported by Aura and Härkönen [108]. In brief, dissolved tea (5 mL) was first subjected to the oral digestion phase by mixing it with 15 mL of milli-Q water, 10 mL of 0.85% NaCl, and salivary amylase solution (50 U), with the pH adjusted to 6.9. At this phase, the mixture was shaken on an orbital shaker (120 rpm) and incubated at 37 °C for 5 min. Then, the entire amount of the oral bolus was mixed with 4.5 mL of 150 mM HCl and 1 mL of pepsin (2 mg/mL). In the gastric phase, the pH of all mixtures was adjusted to 2.5, with continuous mixing at 37 °C for 2 h. After this time, the gastric chyme of all samples was mixed with 4 mL of bile acids (deoxycholic and cholic acid), 4 mL of pancreatin solution (18.75 mg/mL), and 1 mL of mucins, and the pH was immediately adjusted to 7.0 in all samples. In the intestinal phase, the mixtures were incubated at 37 °C for 3 h. At the end of digestion, milli-Q water was added to all mixtures to achieve a final volume of 45 mL. Thereafter, the digested mixtures were centrifuged (4000 rpm, 10 min), while the supernatants obtained were filtered through 0.45 µm filters (digeste) (samples were labeled as DPT, DPP, and DPO). Dissolved lyophilized tea (5 mL) mixed with milli-Q water to a final volume of 45 mL was used as a control for digestion (labeled as PTc, PPc, and POc). The control samples and digested samples were passed through SPE cartridges and 0.22 µm filters, to prepare for UHPLC Q-ToF MS analysis. The SPE cartridge was conditioned by washing with 5 mL of acidified methyl alcohol and milli-Q water, while the absorbed bioactive compounds were eluted with 1.5 mL of acidified methyl alcohol (0.1% methyl alcohol).

#### UHPLC Q-ToF MS Analysis Digested Samples

Separation and identification of bioactive compounds were performed on an Agilent 1290 Infinity ultra-high-performance liquid chromatography (UHPLC) system coupled to a quadrupole time-of-flight mass spectrometer (6530C Q-ToF-MS) (Agilent Technologies, Inc., Stevens Creek Blvd, Santa Clara, CA, USA), as previously described in detail by Kostić et al. [109]. Spectra were recorded in the *m*/*z* range from 100 to 1700 in both operating ionization modes (ESI+/ESI−), using auto-MS/MS acquisition. Agilent MassHunter software (version B.10.00)was used for instrument control and data analysis. Bioactive compounds were identified based on their exact *m*/*z* mass, MS fragmentation (Table S2), and date from the literature [2,22,24,29,45].

The bioaccessibility of the individual and total identified bioactive compounds (BCs) was presented as the percentage recovery (%) and calculated according to the following Equation (3):

(3) $$Recovery\;\left(\%\right)=\frac{\sum BCd}{\sum BCc}\times 100$$

where *BCd* is the area of each identified compound and sum of identified compounds in the digested tea samples, and *BCc* is the area of each identified compound and sum of identified compounds in the control teas.

The relative content (%) of bioactive compounds in each sample (before and after *in vitro* GID) was calculated as the ratio of the areas of individual and total BCs identified (the percentage of total area is defined as 100% for each sample—Equation (4)).

(4) $$Relative\;content\;\left(\%\right)=\frac{\sum Area\;of\;individual\;BC}{\sum Total\;area\;of\;all\;identified\;BCs}\times 100$$

### 3.10. Statistical Analysis

With the aim of investigating the statistical significance of the factors’ influence (locality and extraction method) and the interaction between factors, as well as the combination of the factors for obtaining the highest TPC in all employed *Paeonia* species, the statistical analysis was performed employing one-way analysis of variance (ANOVA), Duncan’s post hoc test, and experimental design (software STATISTIC 7.0.). The results are presented as mean value ± standard deviation (except in the case of antibacterial analysis), and the differences were considered statistically significant at *p* < 0.05, *n* = 3 (one-way ANOVA and Duncan’s post hoc test). The antibacterial analysis was also performed in triplicate, but the highest value obtained was taken as MIC and MBC (“stricter criteria” rule was applied, which is common in antimicrobial assays). 

## 4. Conclusions

This study offered insight into the chemical profile and *in vitro* biological activities of water–ethanolic extracts of the roots of PT, PP, and PO wild plants growing at different localities in Serbia. The results of chemical characterization show that *Paeonia* terpenes are the most dominant class of molecules presented in root extracts; hot water extracts are the source of different classes of bioactives, such as gallic and ellagic acid derivatives, flavonoids, procyanidins, and *Paeonia* root terpenoids. Among the tested root extracts, the highest TPC was observed in the PP extract obtained by MAE (Pirot) and in the PO extract obtained by method M (Rujevica). Antioxidant activity varied depending on the species and origin of the tested peony roots, as well as the extraction method implemented. According to that, the results showed that the highest ABTS scavenging potential was obtained in the PT extracts from Pančevo (MAE). The antioxidant activity of the extracts measured by the DPPH method also varied, depending on both the locality and extraction method. The extracts show various enzyme-inhibitory potentials on both AChE/BuChE, HPA, HIG, and tyrosinase, being the most effective in the inhibition of the AChE/BuChE enzymes. On the other hand, all root extracts show very similar and moderate antibacterial activity against two Gram-positive (*B. cereus* and *L. monocytogenes*) and one Gram-negative (*P. aeruginosa*) bacteria strains; the most effective samples were macerates of PP (Južni Kučaj) and PO (Rujevica). The molecular docking analysis on five enzymes provided a detailed overview of the protein–ligand interactions contributing to the observed activities of the compounds from *Peaonia* root extracts. In addition, the GID results showed the dominant presence of methyl gallate and digallate in digests, probably originating from tannin derivatives, which may have potentially strong activity, making the hot water extracts (teas) eligible for use as food supplements. 

In summary, the PT, PP, and PO extracts, as well as hot water extracts (teas), proved to be great sources of bioactives due to their unique chemical composition and demonstrated biological potential, which makes them promising constituents to include in many novel products of the pharmaceutical, cosmetic, and food industries. In most cases, PT from Gulenovci, PP from Pirot, and PO from Rujevica have strong activity in the assays used. The results of antioxidant, enzyme-inhibitory, and antibacterial activities, as well as the content of the extracted polyphenols, varied depending on the extraction method used, and they showed that the use of MAE is the most beneficial for the extraction of bioactive molecules from PP root extracts. In the analyzed extracts of the roots of the other two *Paoenia* species, UAE and method M alternated, without favoring either one. The mentioned plant resources could be highly recommended for further cultivation, which will enable the continuous supply of industries with herbal raw materials (the roots) of standardized quality.

## Figures and Tables

**Table 2:** Total polyphenol content (TPC) of the root extracts of *Paeonia tenuifolia* L., *Paeonia peregrina* Mill., and *Paeonia officinalis* L.

Plant Species (Root Extract)	Locality	Extraction Method	TPC [mg GAE/g]
PT	Gulenovci	MAE	77.20 ± 2.56
	Deliblato sand		67.64 ± 0.75
	Bogovo gumno		31.14 ± 1.38
	Gulenovci	UAE	87.89 ± 1.38
	Deliblato sand		99.64 ± 0.25
	Bogovo gumno		36.45 ± 3.94
	Gulenovci	M	53.70 ± 2.94
	Deliblato sand		47.76 ± 1.00
	Bogovo gumno		72.58 ± 2.56
PP	Pirot	MAE	161.2 ± 2.19
	Južni Kučaj		110.7 ± 4.63
	Golina		82.20 ± 3.94
	Bogovo gumno		72.45 ± 0.94
	Krivi vir		48.20 ± 0.81
	Pančevo		91.14 ± 4.63
	Pirot	UAE	93.58 ± 0.56
	Južni Kučaj		32.39 ± 0.13
	Golina		57.01 ± 6.63
	Bogovo gumno		43.20 ± 4.19
	Krivi vir		69.01 ± 0.63
	Pančevo		143.7 ± 2.56
	Pirot	M	105.9 ± 15.8
	Južni Kučaj		81.01 ± 5.63
	Golina		83.89 ± 6.13
	Bogovo gumno		54.26 ± 0.00
	Krivi vir		95.08 ± 1.06
	Pančevo		109.3 ± 3.06
PO	Rujevica	MAE	35.01 ± 2.50
	Božurna		82.58 ± 8.56
	Rujevica	UAE	49.26 ± 2.63
	Božurna		77.58 ± 3.44
	Rujevica	M	165.6 ± 5.00
	Božurna		101.2 ± 3.81

MAE: microwave-assisted extraction; UAE: ultrasound-assisted extraction; M: maceration; PT: *Paeonia tenuifolia* L.; PP: *Paeonia peregrina* Mill.; PO: *Paeonia officinalis* L.; GAE: gallic acid equivalents.

**Table 3:** Statistical analysis of optimization of polyphenol extraction of roots from three *Paeonia* species (*Paeonia tenuifolia* L., *Paeonia peregrina* Mill., and *Paeonia officinalis* L.) collected in different localities and employing maceration and microwave- and ultrasound-assisted extractions.

	TPC [mg GAE/g]	Effect.	Std. Err.	Effect Estimates	Coeff.	Std. Err. Coeff.	*p*
PT	Constant				82.910	0.449	0.000
	Locality (1)	1.267	0.898	1.410	0.633	0.449	0.196
	Extraction procedure (2)	−21.527	0.898	−23.965	−10.763	0.449	0.000
	1 by 2	−10.573	0.898	−11.771	−5.287	0.449	0.000
PP	Constant				123.727	1.530	0.000
	Locality (1)	13.342	3.061	4.358	6.671	1.530	0.002
	Extraction procedure (2)	56.908	3.061	18.591	28.454	1.530	0.000
	1 by 2	4.555	3.061	1.488	2.277	1.530	0.175
PO	Constant				71.032	0.417	0.000
	Locality (1)	41.532	0.835	49.731	20.766	0.417	0.000
	Extraction procedure (2)	24.535	0.835	29.379	12.267	0.417	0.000
	1 by 2	−5.998	0.835	−7.182	−2.999	0.417	0.000

TPC: total polyphenol content; PT: *Paeonia tenuifolia* L.; PP: *Paeonia peregrina* Mill.; PO: *Paeonia officinalis* L.; GAE: gallic acid equivalents.

**Table 4:** Total flavonoid content of the root extracts of *Paeonia tenuifolia* L., *Paeonia peregrina* Mill., and *Paeonia officinalis* L.

Plant Species (Root Extract)	Locality	Extraction Method	TFC [mg CE/g]
PT	Gulenovci	MAE	2.30 ± 0.38 ^d^
	Deliblato sand		1.84 ± 0.28 ^de^
	Bogovo gumno		0.94 ± 0.45 ^fg^
	Gulenovci	UAE	2.97 ± 0.85 ^d^
	Deliblato sand		3.27 ± 0.55 ^cd^
	Bogovo gumno		1.09 ± 0.27 ^f^
	Gulenovci	M	1.29 ± 0.23 ^f^
	Deliblato sand		0.99 ± 0.50 ^fg^
	Bogovo gumno		2.24 ± 0.12 ^d^
PP	Pirot	MAE	5.75 ± 0.37 ^ab^
	Južni Kučaj		4.10 ± 0.35 ^c^
	Golina		2.34 ± 0.28 ^d^
	Bogovo gumno		2.14 ± 0.22 ^d^
	Krivi vir		1.10 ± 0.17 ^fg^
	Pančevo		2.99 ± 0.42 ^d^
	Pirot	UAE	3.03 ± 0.65 ^d^
	Južni Kučaj		0.99 ± 0.15 ^gh^
	Golina		1.53 ± 0.10 ^ef^
	Bogovo gumno		0.74 ± 0.15 ^h^
	Krivi vir		1.93 ± 0.30 ^e^
	Pančevo		5.35 ± 0.03 ^b^
	Pirot	M	3.99 ± 0.53 ^cd^
	Južni Kučaj		2.14 ± 0.85 ^d^
	Golina		2.32 ± 0.13 ^d^
	Bogovo gumno		1.37 ± 0.18 ^f^
	Krivi vir		3.14 ± 0.65 ^cd^
	Pančevo		4.00 ± 0.92 ^c^
PO	Rujevica	MAE	1.01 ± 0.10 ^g^
	Božurna		2.39 ± 0.15 ^d^
	Rujevica	UAE	1.01 ± 0.06 ^g^
	Božurna		2.39 ± 0.07 ^d^
	Rujevica	M	5.92 ± 0.33 ^a^
	Božurna		3.72 ± 0.23 ^cd^

MAE: microwave-assisted extraction; UAE: ultrasound-assisted extraction; M: maceration; CE: catechin equivalents; PT: *Paeonia tenuifolia* L.; PP: *Paeonia peregrina* Mill.; PO: *Paeonia officinalis* L.; TFC: total flavonoid content; values with the same letter in the column showed no statistically significant difference (*p* > 0.05; *n* = 3, one-way ANOVA, analysis of variance, Duncan’s post hoc test).

**Table 5:** Antioxidant activity of root extracts of *Paeonia tenuifolia* L., *Paeonia peregrina* Mill., and *Paeonia officinalis* L. obtained by different extraction methods.

Plant Species (Root Extract)	Locality	Extraction Method	Antioxidant Activity	
			ABTS [µg TE/mL]	DPPH [IC_50_, mg/mL]
PT	Gulenovci	MAE	15.0 ± 0.5 ^e^	0.207 ± 0.054 ^ab^
	Deliblato sand		8.0 ± 0.2 ^j^	0.203 ± 0.022 ^b^
	Bogovo gumno		15.0 ± 0.2 ^e^	0.250 ± 0.000 ^c^
	Gulenovci	UAE	19.5 ± 0.5 ^bc^	0.179 ± 0.052 ^ab^
	Deliblato sand		12.0 ± 1.0 ^fg^	0.168 ± 0.056 ^ab^
	Bogovo gumno		8.0 ± 0.5 ^ij^	0.199 ± 0.048 ^ab^
	Gulenovci	M	28.5 ± 1.8 ^a^	0.190 ± 0.0843 ^ab^
	Deliblato sand		10.5 ± 0.8 ^gh^	0.188 ± 0.055 ^ab^
	Bogovo gumno		13.0 ± 0.9 ^f^	0.184 ± 0.034 ^ab^
PP	Pirot	MAE	8.5 ± 0.5 ^ij^	0.221 ± 0.014 ^b^
	Južni Kučaj		10.5 ± 0. 5 ^gh^	0.188 ± 0.039 ^ab^
	Golina		6.0 ± 0.5 ^k^	0.244 ± 0.068 ^bc^
	Bogovo gumno		9.0 ± 0.5 ^i^	0.235 ± 0.037 ^bc^
	Krivi vir		18.0 ± 1.5 ^bc^	0.266 ± 0.047 ^bc^
	Pančevo		30.0 ± 1.0 ^a^	0.196 ± 0.037 ^ab^
	Pirot	UAE	12.0 ± 0.5 ^g^	0.186 ± 0.056 ^ab^
	Južni Kučaj		8.5 ± 0.5 ^ij^	0.190 ± 0.019 ^ab^
	Golina		16.5 ± 0.5 ^cd^	0.269 ± 0.048 ^bc^
	Bogovo gumno		14.0 ± 1.0 ^ef^	0.235 ± 0.034 ^bc^
	Krivi vir		15.0 ± 1.0 ^de^	0.224 ± 0.014 ^bc^
	Pančevo		11.5 ± 0.5 ^g^	0.186 ± 0.044 ^ab^
	Pirot	M	13.5 ± 0.5 ^f^	0.191 ± 0.033 ^ab^
	Južni Kučaj		10.5 ± 0.9 ^gh^	0.187 ± 0.057 ^ab^
	Golina		11.0 ± 0.7 ^g^	0.233 ± 0.039 ^bc^
	Bogovo gumno		20.5 ± 0.8 ^b^	0.171 ± 0.058 ^ab^
	Krivi vir		16.0 ± 0.3 ^d^	0.199 ± 0.059 ^ab^
	Pančevo		18.5 ± 0.9 ^c^	0.165 ± 0.055 ^ab^
PO	Rujevica	MAE	13.0 ± 0.5 ^c^	0.170 ± 0.039 ^ab^
	Božurna		10.5 ± 0. 5 ^gh^	0.207 ± 0.015 ^b^
	Rujevica	UAE	10.5 ± 0.7 ^gh^	0.190 ± 0.032 ^ab^
	Božurna		20.0 ± 1.0 ^bc^	0.213 ± 0.021 ^b^
	Rujevica	M	19.5 ± 0.8 ^bc^	0.168 ± 0.054 ^ab^
	Božurna		12.0 ± 0.8 ^g^	0.145 ± 0.0350 ^a^

MAE: microwave-assisted extraction; UAE: ultrasound-assisted extraction; M: maceration; PT: *Paeonia tenuifolia* L.; PP: *Paeonia peregrina* Mill.; PO: *Paeonia officinalis* L.; ABTS: 2,2′-azino-bis(3-ethylbenzothiazoline-6-sulfonic acid); DPPH: 2,2-diphenyl-1-picrylhydrazyl; TE: Trolox equivalent; IC_50_: the concentration of the root extract required to neutralize 50% of DPPH^•^ radicals; values with the same letter in each column showed no statistically significant difference (*p* > 0.05; *n* = 3, one-way ANOVA, analysis of variance, Duncan’s post hoc test).

**Table 6:** Enzyme-inhibitory activity of the root extracts of *Paeonia tenuifolia* L., *Paeonia peregrina* Mill., and *Paeonia officinalis* L.

Plant Species (Root Extract)	Locality	Extraction Method	Ache Inhibition	Buche Inhibition	HPA Inhibition	HIG Inhibition	Tyrosinase Inhibition
			[mg GALAE/g]		[mmol ACAE/g]		[mg KAE/g]
PT	Gulenovci	MAE	1.14 ± 0.02 ^j^	0.90 ± 0.14 ^cd^	0.24 ± 0.00 ^b^	1.21 ± 0.01 ^f^	24.02 ± 0.66 ^e^
	Deliblato sand		1.78 ± 0.07 ^cd^	1.41 ± 0.18 ^b^	0.17 ± 0.01 ^f^	1.02 ± 0.02 ^j^	28.73 ± 2.26 ^cd^
	Bogovo gumno		2.43 ± 0.03 ^a^	2.94 ± 0.23 ^a^	0.15 ± 0.01 ^g^	1.23 ± 0.01 ^f^	24.29 ± 0.55 ^e^
	Gulenovci	UAE	1.35 ± 0.02 ^h^	1.01 ± 0.19 ^c^	0.22 ± 0.00 ^c^	1.28 ± 0.01 ^bc^	29.59 ± 2.36 ^c^
	Deliblato sand		1.81 ± 0.05 ^c^	0.78 ± 0.06 ^de^	0.20 ± 0.00 ^e^	1.19 ± 0.01 ^g^	29.99 ± 1.76 ^c^
	Bogovo gumno		1.62 ± 0.02 ^e^	0.98 ± 0.12 ^cd^	0.18 ± 0.00 ^f^	1.13 ± 0.02 ^h^	24.83 ± 1.57 ^e^
	Gulenovci	M	1.22 ± 0.06 ^i^	0.96 ± 0.06 ^cd^	0.24 ± 0.00 ^b^	1.28 ± 0.01 ^bc^	28.63 ± 0.51 ^d^
	Deliblato sand		1.92 ± 0.07 ^bc^	1.09 ± 0.07 ^c^	0.16 ± 0.00 ^fg^	1.16 ± 0.03 ^g^	32.68 ± 1.33 ^b^
	Bogovo gumno		1.70 ± 0.09 ^de^	1.14 ± 0.04 ^c^	0.15 ± 0.01 ^g^	1.09 ± 0.01 ^i^	24.12 ± 1.38 ^e^
PP	Pirot	MAE	1.63 ± 0.02 ^e^	1.01 ± 0.04 ^c^	0.15 ± 0.01 ^g^	1.25 ± 0.00 ^f^	31.05 ± 1.63 ^bc^
	Južni Kučaj		1.69 ± 0.06 ^de^	1.18 ± 0.28 ^bc^	0.18 ± 0.01 ^ef^	1.29 ± 0.00 ^b^	32.34 ± 1.05 ^b^
	Golina		1.25 ± 0.03 ^i^	1.10 ± 0.08 ^c^	0.18 ± 0.00 ^f^	0.62 ± 0.01 ^m^	25.37 ± 2.05 ^de^
	Bogovo gumno		1.41 ± 0.02 ^g^	1.05 ± 0.20 ^c^	0.20 ± 0.01 ^de^	1.22 ± 0.02 ^ef^	26.07 ± 1.32 ^de^
	Krivi vir		0.48 ± 0.08 ^k^	1.11 ± 0.14 ^c^	0.16 ± 0.01 ^fg^	0.90 ± 0.04 ^k^	22.95 ± 1.43 ^f^
	Pančevo		1.35 ± 0.04 ^gh^	1.14 ± 0.17 ^c^	0.21 ± 0.00 ^d^	1.27 ± 0.01 ^cd^	25.68 ± 0.22 ^e^
	Pirot	UAE	1.67 ± 0.05 ^e^	0.68 ± 0.06 ^e^	0.18 ± 0.00 ^f^	1.29 ± 0.00 ^b^	33.99 ± 0.48 ^b^
	Južni Kučaj		1.63 ± 0.03 ^e^	0.74 ± 0.07 ^de^	0.25 ± 0.02 ^a^	1.29 ± 0.00 ^b^	35.50 ± 1.91 ^ab^
	Golina		1.23 ± 0.02 ^i^	0.74 ± 0.07 ^de^	0.24 ± 0.01 ^ab^	0.71 ± 0.06 ^l^	22.18 ± 0.28 ^f^
	Bogovo gumno		1.53 ± 0.04 ^f^	0.67 ± 0.05 ^e^	0.21 ± 0.00 ^d^	1.24 ± 0.02 ^ef^	25.36 ± 1.43 ^e^
	Krivi vir		1.82 ± 0.04 ^c^	1.03 ± 0.19 ^c^	0.23 ± 0.01 ^b^	1.03 ± 0.02 ^j^	27.44 ± 0.77 ^d^
	Pančevo		1.54 ± 0.03 ^f^	1.26 ± 0.16 ^bc^	0.18 ± 0.00 ^f^	1.14 ± 0.03 ^h^	30.22 ± 0.68 ^c^
	Pirot	M	2.00 ± 0.01 ^b^	1.38 ± 0.03 ^b^	0.20 ± 0.00 ^e^	1.28 ± 0.00 ^c^	35.33 ± 1.71 ^ab^
	Južni Kučaj		1.73 ± 0.02 ^d^	1.17 ± 0.04 ^c^	0.23 ± 0.01 ^b^	1.29 ± 0.00 ^b^	30.57 ± 0.60 ^c^
	Golina		1.31 ± 0.02 ^h^	0.81 ± 0.02 ^d^	0.18 ± 0.00 ^f^	0.66 ± 0.05 ^lm^	23.28 ± 1.22 ^ef^
	Bogovo gumno		1.40 ± 0.01 ^g^	0.82 ± 0.05 ^d^	0.26 ± 0.01 ^a^	1.28 ± 0.01 ^bc^	27.61 ± 2.67 ^cd^
	Krivi vir		1.42 ± 0.02 ^g^	1.10 ± 0.04 ^c^	0.20 ± 0.01 ^de^	1.01 ± 0.01 ^j^	23.16 ± 1.16 ^ef^
	Pančevo		1.46 ± 0.08 ^fg^	1.10 ± 0.08 ^c^	0.19 ± 0.01 ^ef^	1.26 ± 0.00 ^e^	27.97 ± 2.13 ^cd^
PO	Rujevica	MAE	1.69 ± 0.02 ^de^	1.21 ± 0.09 ^bc^	0.23 ± 0.01 ^b^	1.26 ± 0.00 ^e^	33.67 ± 1.19 ^b^
	Božurna		1.65 ± 0.05 ^de^	0.79 ± 0.06 ^d^	0.21 ± 0.00 ^d^	1.27 ± 0.00 ^d^	30.08 ± 0.90 ^c^
	Rujevica	UAE	1.85 ± 0.06 ^c^	1.42 ± 0.17 ^b^	0.24 ± 0.00 ^b^	1.29 ± 0.00 ^b^	35.96 ± 1.05 ^ab^
	Božurna		1.82 ± 0.03 ^c^	0.66 ± 0.04 ^e^	0.21 ± 0.00 ^d^	1.29 ± 0.00 ^b^	30.44 ± 1.18 ^c^
	Rujevica	M	1.72 ± 0.02 ^d^	1.34 ± 0.07 ^b^	0.24 ± 0.01 ^ab^	1.30 ± 0.00 ^a^	37.06 ± 1.33 ^a^
	Božurna		1.62 ± 0.03 ^e^	1.20 ± 0.09 ^bc^	0.20 ± 0.00 ^e^	1.28 ± 0.00 ^c^	26.80 ± 0.63 ^d^

GALAE: galanthamine equivalents; ACAE: acarbose equivalents; KAE: kojic acid equivalents; AChE: acetylcholinesterase; BuChE: butyrylcholinesterase; HPA: human pancreatic α-amylase; HIG: human intestinal α-glycosidase; PT: *Paeonia tenuifolia* L.; PP: *Paeonia peregrina* Mill.; PO: *Paeonia officinalis* L.; values with the same letter in each column showed no statistically significant difference (*p* > 0.05; *n* = 3, one-way ANOVA, analysis of variance, Duncan’s post hoc test); MAE: microwave-assisted extraction; UAE: ultrasound-assisted extraction; M: maceration.

**Table 7:** Antibacterial activity of the root extracts of *Paeonia tenuifolia* L., *Paeonia peregrina* Mill., and *Paeonia officinalis* L. expressed as MIC and MBC (mg/mL).

Plant Species (Root Extract)	Locality	Extraction Method	*Salmonella* Typhimurium		*Listeria monocytogenes*		*Bacillus cereus*		*Pseudomonas aeruginosa*		*Staphylococcus aureus*		*Escherichia coli*	
			MIC	MBC	MIC	MBC	MIC	MBC	MIC	MBC	MIC	MBC	MIC	MBC
PT	Gulenovci	MAE	2.0	4.0	4.0	8.0	2.0	4.0	4.0	8.0	4.0	8.0	2.0	4.0
	Deliblato sand		1.0	2.0	2.0	4.0	2.0	4.0	2.0	4.0	2.0	4.0	2.0	4.0
	Bogovo gumno		4.0	8.0	4.0	8.0	4.0	8.0	4.0	8.0	4.0	8.0	2.0	4.0
	Gulenovci	UAE	1.0	2.0	1.0	2.0	0.5	1.0	1.0	2.0	2.0	4.0	1.0	2.0
	Deliblato sand		2.0	4.0	1.0	2.0	1.0	2.0	2.0	4.0	2.0	4.0	1.0	2.0
	Bogovo gumno		0.5	1.0	1.0	2.0	1.0	2.0	0.5	1.0	2.0	4.0	1.0	2.0
	Gulenovci	M	1.0	2.0	2.0	4.0	1.0	2.0	1.0	2.0	1.0	2.0	1.0	2.0
	Deliblato sand		1.0	2.0	1.0	2.0	1.0	2.0	1.0	2.0	2.0	4.0	1.0	2.0
	Bogovo gumno		2.0	4.0	1.0	2.0	1.0	2.0	1.0	2.0	2.0	4.0	0.5	1.0
PP	Pirot	MAE	4.0	8.0	4.0	8.0	4.0	8.0	4.0	8.0	4.0	8.0	4.0	8.0
	Južni Kučaj		2.0	4.0	2.0	4.0	1.0	2.0	2.0	4.0	2.0	4.0	1.0	2.0
	Golina		2.0	4.0	2.0	4.0	1.0	2.0	1.0	2.0	2.0	4.0	1.0	2.0
	Bogovo gumno		4.0	8.0	4.0	8.0	2.0	4.0	4.0	8.0	2.0	4.0	2.0	4.0
	Krivi vir		2.0	4.0	2.0	4.0	2.0	4.0	1.0	2.0	2.0	4.0	2.0	4.0
	Pančevo		4.0	8.0	2.0	4.0	1.0	2.0	2.0	4.0	2.0	4.0	0.5	1.0
	Pirot	UAE	2.0	4.0	1.0	2.0	1.0	2.0	2.0	4.0	2.0	4.0	1.0	2.0
	Južni Kučaj		1.0	2.0	1.0	2.0	1.0	2.0	1.0	2.0	1.0	2.0	2.0	4.0
	Golina		4.0	8.0	2.0	4.0	1.0	2.0	2.0	4.0	2.0	4.0	1.0	2.0
	Bogovo gumno		1.0	2.0	1.0	2.0	1.0	2.0	1.0	2.0	2.0	4.0	1.0	2.0
	Krivi vir		4.0	8.0	1.0	2.0	1.0	2.0	2.0	4.0	4.0	8.0	1.0	2.0
	Pančevo		1.0	2.0	1.0	2.0	2.0	4.0	0.5	1.0	2.0	4.0	2.0	4.0
	Pirot	M	1.0	2.0	0.5	1.0	0.5	1.0	1.0	2.0	1.0	2.0	1.0	2.0
	Južni Kučaj		1.0	2.0	1.0	2.0	0.125	0.25	1.0	2.0	1.0	2.0	0.5	1.0
	Golina		1.0	2.0	1.0	2.0	0.25	0.5	1.0	2.0	1.0	2.0	0.5	1.0
	Bogovo gumno		1.0	2.0	0.5	1.0	1.0	2.0	0.5	1.0	2.0	4.0	1.0	2.0
	Krivi vir		0.5	1.0	1.0	2.0	0.25	0.5	0.5	1.0	0.5	1.0	0.5	1.0
	Pančevo		1.0	2.0	2.0	4.0	1.0	2.0	1.0	2.0	2.0	4.0	0.5	1.0
PO	Rujevica	MAE	2.0	4.0	2.0	4.0	2.0	4.0	2.0	4.0	2.0	4.0	2.0	4.0
	Božurna		2.0	4.0	1.0	2.0	0.5	1.0	2.0	4.0	2.0	4.0	0.5	1.0
	Rujevica	UAE	2.0	4.0	1.0	2.0	0.5	1.0	0.5	1.0	2.0	4.0	1.0	2.0
	Božurna		1.0	2.0	1.0	2.0	0.5	1.0	0.5	1.0	2.0	4.0	1.0	2.0
	Rujevica	M	1.0	2.0	0.25	0.5	0.5	1.0	0.25	0.5	1.0	2.0	0.5	1.0
	Božurna		1.0	2.0	1.0	2.0	0.5	1.0	0.5	1.0	1.0	2.0	0.5	1.0

MAE: microwave-assisted extraction; UAE: ultrasound-assisted extraction; M: maceration; MIC: minimal inhibitory concentration; MBC: minimal bactericidal concentration; GALAE: galanthamine equivalents; ACAE: acarbose equivalents; KAE: kojic acid equivalents; AChE: acetylcholinesterase; BuChE: butyrylcholinesterase; PT: *Paeonia tenuifolia* L.; PP: *Paeonia peregrina* Mill.; PO: *Paeonia officinalis* L. The antibacterial study was repeated three times (*n* = 3); the highest values obtained in the test were used as MIC and MBC (like with most antimicrobial tests, the “stricter criteria” rule was applied).

**Table 8:** Relative content of identified bioactive compounds (%) in tea samples before and after GID using UHPLC Q-ToF MS.

No.	Compound Name	Relative Content (%) *					
		PTc	DPT	PPc	DPP	POc	DPO
Phenolic acids and derivatives							
Gallic acid and derivatives							
1	Gallic acid	0.69	0.27	1.10	0.19	2.24	0.38
2	Methyl gallate	0.09	7.14	0.18	3.80	2.34	21.30
3	Gallic acid derivative	0.79	0.14	0.14	0.03	1.05	0.08
4	Digallic acid isomer I	0.21	0.05	-	-	1.00	-
5	Digallic acid isomer II	0.52	0.09	0.19	-	1.58	0.05
6	Gallic acid hexoside	1.21	0.62	1.62	0.62	1.54	0.41
7	Methyl digallate	0.22	1.21	-	0.11	0.03	1.70
8	Methyl gallic acid hexoside	0.06	-	0.34	0.06	0.05	-
9	Galloyl-vanilloyl-rhamoside	0.31	0.11	0.06	-	2.82	1.57
10	Gallic acid dihexoside	7.98	2.44	4.26	1.21	13.70	5.01
11	Galloyl-HHDP-hexose isomer II	0.97	0.11	0.44	0.04	1.58	0.15
12	Galloyl-HHDP-hexose isomer I	0.78	0.10	0.50	-	1.21	0.08
13	Trigalloyl-hexoside	0.33	0.08	0.26	0.09	-	-
14	Digalloyl-HHDP-protoquercitol	0.14	-	0.20	0.12	0.75	0.53
15	Tetragalloyl-hexoside	0.17	0.12	0.35	0.19	1.31	0.61
16	Pentagalloyl-hexoside	0.39	0.33	0.43	0.30	2.40	1.50
∑		14.66	12.83	10.07	6.64	33.59	33.38
Ellagic acid and derivatives							
17	Ellagic acid	0.83	0.29	2.37	0.73	4.18	1.53
18	Methyl ellagic acid	0.41	0.19	0.80	0.28	1.25	0.43
19	Dimethyl ellagic acid	0.13	0.04	0.20	0.19	1.19	1.49
20	Trimethyl ellagic acid	-	-	1.48	1.12	0.06	0.07
21	Ellagic acid hexoside	0.35	0.03	0.45	0.06	0.82	0.11
22	Methyl ellagic acid hexoside	0.46	0.18	0.28	0.12	0.52	0.22
∑		2.18	0.73	5.58	2.50	8.03	3.86
Other phenolic acids and their glycosides							
23	Hydroxybenzoic acid	1.26	0.84	1.46	1.10	1.98	1.33
24	Dihydroxybenzoic acid isomer I	0.38	-	0.50	-	0.29	-
25	Dihydroxybenzoic acid isomer II	0.15	0.0.04	-	-	0.45	0.25
26	Dihydroxybenzoic acid hexoside	0.23	0.18	0.45	0.27	0.77	0.25
27	Vanillic acid hexoside	0.39	0.10	1.12	0.33	0.16	-
∑		2.42	1.16	3.53	1.70	3.64	1.83
∑RC of total phenolic acid		19.26	14.71	19.18	10.84	45.26	39.06
Flavonoids and derivatives							
Flavan-3-ols and procyanidins							
28	Catechin	13.86	12.97	16.28	12.58	5.08	2.57
29	Epicatechin	1.10	0.74	2.57	1.74	0.65	0.27
30	Methyl epigallocatechin	0.14	0.02	0.13	-	0.12	-
31	Epicatechin-gallate	0.27	0.21	0.16	-	0.04	-
32	Catechin hexoside	0.81	0.56	3.92	3.66	4.35	3.00
33	Epicatechin-hexoside	0.38	0.22	0.55	0.41	0.21	-
34	B-type procyanidin dimer isomer I	22.20	18.55	8.88	1.94	6.02	2.35
35	B-type procyanidin dimer isomer II	1.12	0.82	2.58	2.35	0.67	0.43
36	Chalcan flavan-3-ols dimer isomer II	0.20	0.16	0.87	0.50	-	-
37	Chalcan flavan-3-ol dimer isomer I	6.41	6.08	7.11	4.88	0.71	0.23
38	Methyl B-type prodelphinidin	0.27	-	0.26	-	0.21	-
39	Procyanidin trimer B-type isomer I	2.48	1.85	2.72	2.39	0.50	0.38
40	Procyanidin trimer B-type isomer II	0.58	0.18	0.37	-	0.08	-
∑		49.82	42.35	46.39	42.02	18.65	9.24
Other detected flavonoids							
41	Kaempferol	0.06	-	0.08	0.04	0.04	0.02
42	Naringenin	0.07	0.07	0.12	0.12	0.14	0.17
43	Phloridzin	0.26	0.33	0.36	0.45	1.25	1.22
∑		0.39	0.40	0.56	0.61	1.43	1.41
∑RC of total flavonoids		50.21	42.75	46.94	31.06	20.07	10.65
Peonia root terpenoids							
44	Nor-paeonilactone	-	-	0.08	0.03	0.18	-
45	Paeoveitol D	0.91	0.58	0.71	-	0.64	-
46	Paeonisothujone	0.57	0.03	0.44	-	1.44	-
47	Paeonilactone B	0.34	0.09	0.46	0.21	0.93	0.23
48	Paeonilactone A	-	-	0.39	0.22	1.73	-
49	9-Hydroxypaeonilactone A	0.09	-	2.55	0.67	1.07	-
50	Paeoniflorigenone	0.31	0.18	4.22	7.18	2.66	1.96
51	Oxypaeoniflorin	6.18	5.53	4.81	4.14	5.02	4.66
52	Mudanpioside D	-	-	0.23	0.22	-	-
53	Paeoniflorin + HCOOH	12.91	8.61	14.17	13.28	15.72	10.24
54	Albiflorin + HCOOH	3.64	4.36	3.29	3.40	1.96	2.19
55	Galloyl desbenzoyl paeoniflorin	0.82	0.39	0.84	0.26	-	-
56	Benzoyl paeoniflorin	-	-	-	-	0.21	0.11
57	Mudanpioside J	1.03	1.10	0.26	0.24	1.39	1.30
58	Galloyl-paeoniflorin	3.74	3.58	1.44	0.73	1.72	1.32
∑RC of total terpenoids		30.53	24.45	33.88	30.58	34.66	22.03
		100	-	100	-	100	-
**		100	-	92.37	-	79.00	-

GID: digestion in the gastrointestinal tract; * The relative content (%) of bioactive compounds in each sample (before and after gastrointestinal digestion) (share of total area is defined as 100% for each sample). ** Comparison of the share of total identified bioactive compounds among tea samples. “-” nonidentified compounds. DPT: digested of *Paeonia tenuifolia* tea; PTc: control (initial) *Paeonia tenuifolia* tea; DPP: digested of *Paeonia peregrina* tea; PPc: control (initial) *Paeonia peregrina* tea; DPO: digested of *Paeonia officinalis* tea; POc: control (initial) *Paeonia officinalis* tea.

**Table 9:** Recovery of total and individual bioactive compounds detected in the selected *Paeonia* root teas after *in vitro* GID using UHPLC Q-ToF MS. The expected retention time (RT), molecular formula, and exact *m*/*z* mass of the confirmed compounds are presented in Table 1.

No	RT	Compound Name	Formula	*m*/*z* Exact Mass	Recovery after In Vitro GID (%)			Recovery Range (%)
					DPT	DPP	DPO	
Phenolic acids and derivatives								
Gallic acid and derivatives								
1	1.14	Gallic acid	C_7_H_5_O_5_^−^	169.01443	39.18	17.11	17.02	17.02–39.18
2	4.24	Methyl gallate	C_8_H_7_O_5_^−^	183.02825	7538.45	2060.68	910.95	910.95–7538.45
3	1.75	Gallic acid derivative	C_10_H_11_O_7_^−^	243.05244	17.62	20.88	7.57	7.57–20.88
4	3.77	Digallic acid isomer I	C_14_H_9_O_9_^−^	321.02654	23.72	-	0	0–23.72
5	5.59	Digallic acid isomer II	C_14_H_9_O_9_^−^	321.02743	17.21	0	3.11	3.11–17.21
6	0.90	Gallic acid hexoside	C_13_H_15_O_10_^−^	331.06817	50.75	38.39	26.64	26.64–50.75
7	8.02	Methyl digallate	C_15_H_11_O_9_^−^	335.04267	5484	**	5686.48	>5484
8	1.75	Methyl gallic acid hexoside	C_14_H_17_O_10_^−^	345.08617	0	16.97	0	0–16.97
9	7.34	Galloyl-vanilloyl-rhamoside	C_21_H_21_O_12_^−^	465.10780	35.54	0	55.84	0–55.84
10	1.00	Gallic acid dihexoside	C_19_H_25_O_15_^−^	493.12260	30.64	28.33	36.54	28.33–36.54
11	5.93	Galloyl-HHDP-hexose isomer II	C_27_H_21_O_18_^−^	633.07382	11.28	9.49	9.24	9.24–11.28
12	2.89	Galloyl-HHDP-hexose isomer I	C_27_H_21_O_18_^−^	633.07577	13.45	0	6.82	0–13.45
13	6.80	Trigalloyl-hexoside	C_27_H_23_O_18_^−^	635.09260	25.11	34.26	-	25.11–34.26
14	8.15	Digalloyl-HHDP-protoquercitol	C_34_H_27_O_21_^+^	771.10856	0	60.06	70.22	0–70.22
15	7.55	Tetragalloyl-hexoside	C_34_H_27_O_22_^−^	787.10066	70.80	54.59	46.96	46.96–70.80
16	7.95	Pentagalloyl-hexoside	C_41_H_31_O_26_^−^	939.11237	85.75	69.30	62.73	62.73–85.75
∑					87.49	65.93	99.37	65.93–99.37
Ellagic acid and derivatives								
17	7.68	Ellagic acid	C_14_H_5_O_8_^−^	301.00097	34.42	31.04	36.58	31.04–36.58
18	8.42	Methyl ellagic acid	C_15_H_7_O_8_^−^	315.01710	46.31	35.33	34.48	34.48–46.31
19	9.57	Dimethyl ellagic acid	C_16_H_9_O_8_^−^	329.03194	31.94	95.14	125.18	31.94–125.18
20	10.85	Trimethyl ellagic acid	C_17_H_11_O_8_^−^	343.04668	-	75.52	111.78	75.52–111.78
21	6.87	Ellagic acid hexoside	C_20_H_15_O_13_^−^	463.05601	9.51	12.21	13.09	9.51–13.09
22	7.55	Methyl ellagic acid hexoside	C_21_H_17_O_13_^−^	477.07058	38.10	43.04	43.03	38.10–43.04
∑					33.32	44.81	48.03	33.32–48.03
Other phenolic acids and their glycosides								
23	3.09	Hydroxybenzoic acid	C_7_H_5_O_3_^−^	137.02422	66.54	75.62	67.07	66.54–75.62
24	1.88	Dihydroxybenzoic acid isomer I	C_7_H_5_O_4_^−^	153.01946	0	0	0	0
25	5.12	Dihydroxybenzoic acid isomer II	C_7_H_5_O_4_^−^	153.01911	26.70	-	56.35	26.70–56.35
26	2.36	Hydroxybenzoic acid hexoside	C_13_H_15_O_9_^−^	315.07520	79.52	59.13	32.25	32.25–79.52
27	2.96	Vanillic acid hexoside	C_14_H_17_O_9_^−^	329.08958	25.02	29.84	0	0–29.84
∑					48.01	48.25	50.19	48.01–50.19
∑ Total recovery of phenolic acid and derivatives					76.40	56.53	86.30	56.53–86.30
Flavonoids and derivatives								
Flavan-3-ols and procyanidins								
28	5.29	Catechin	C_15_H_13_O_6_^−^	289.07260	93.57	77.26	50.61	50.61–93.57
29	6.63	Epicatechin	C_15_H_13_O_6_^−^	289.07259	67.61	67.86	41.95	41.95–67.86
30	6.54	Methyl epigallocatechin	C_16_H_15_O_7_^−^	319.08293	12.38	0	-	0–12.38
31	7.68	Epicatechin-gallate	C_22_H_17_O_10_^−^	441.08941	80.00	0	-	0–80.00
32	4.18	Catechin hexoside	C_21_H_23_O_11_^−^	451.12584	68.83	93.38	69.09	69.09–93.38
33	5.73	Epicatechin-hexoside	C_21_H_25_O_11_^+^	453.14384	56.80	75.10	-	56.80–75.10
34	4.51	B-type procyanidin dimer isomer I	C_30_H_25_O_12_^−^	577.13795	83.56	21.80	39.07	21.80–83.56
35	6.33	B-type procyanidin dimer isomer II	C_30_H_25_O_12_^−^	577.13723	73.12	91.33	63.74	63.74–91.33
36	6.63	Chalcan flavan-3-ols dimer iso. II	C_30_H_27_O_12_^−^	579.15511	76.98	57.41	-	57.41–76.98
37	5.29	Chalcan flavan-3-ol dimer isomer I	C_30_H_27_O_12_^−^	579.15287	94.87	68.67	33.12	33.12–94.87
38	6.19	Methyl B-type prodelphinidin	C_31_H_27_O_13_^−^	607.14904	0	0	-	0
39	6.02	Procyanidin trimer B-type isomer I	C_45_H_37_O_18_^−^	865.19890	74.33	87.76	75.93	74.33–87.76
40	6.93	Procyanidin trimer B-type isomer II	C_45_H_37_O_18_^−^	865.19733	31.38	0	-	0–31.38
∑					85.00	65.65	49.75	49.57–85.00
Other detected flavonoids								
41	10.16	Kaempferol	C_15_H_9_O_6_^−^	285.04097	0	54.56	52.58	0–54.56
42	9.91	Naringenin	C_15_H_11_O_5_^−^	271.06201	102.19	103.78	116.53	102.19–116.53
43	8.49	Phloridzin	C_21_H_23_O_10_^−^	435.12768	128.75	122.32	98.08	98.08–128.75
∑					103.34	109.27	98.78	98.78–109.27
∑ Total recovery of flavonoids and derivatives					85.14	66.16	53.07	53.07–85.14
Paeonia root terpenoids								
44	9.97	Nor-paeonilactone	C_9_H_15_O_2_^+^	155.10880	-	37.72	-	37.72
45	6.40	Paeoveitol D	C_10_H_11_O_3_^+^	179.07217	63.25	0	-	0–63.25
46	6.93	Paeonisothujone	C_10_H_15_O_3_^+^	183.10219	4.85	0	-	0–4.85
47	6.60	Paeonilactone B	C_10_H_13_O_4_^+^	197.08347	27.44	46.58	25.02	25.02–46.58
48	3.50	Paeonilactone A	C_10_H_15_O_4_^+^	199.09852	-	56.27	-	56.27
49	2.62	9-Hydroxypaeonilactone A	C_10_H_15_O_5_^+^	215.09411	0	26.31	-	0–26.31
50	10.31	Paeoniflorigenone	C_17_H_19_O_6_^+^	319.12118	56.97	170.17	73.95	56.97–170.17
51	5.82	Oxypaeoniflorin	C_23_H_27_O_12_^−^	495.15174	89.52	86.15	92.85	86.15–92.85
52	8.90	Mudanpioside D	C_24_H_29_O_12_^−^	509.17478	-	94.92	-	94.92
53	7.07	Paeoniflorin + HCOOH	C_24_H_29_O_13_^−^	525.16398	66.69	93.73	65.16	65.16–93.73
54	7.75	Albiflorin + HCOOH	C_24_H_29_O_13_^−^	525.16361	119.68	103.46	111.85	103.46–119.68
55	2.59	Galloyl desbenzoyl paeoniflorin	C_23_H_27_O_14_^−^	527.14182	47.83	30.60	-	30.60–47.83
56	10.17	Benzoyl paeoniflorin	C_30_H_33_O_12_^+^	585.20263	-	-	53.04	53.04
57	9.91	Mudanpioside J	C_31_H_33_O_14_^−^	629.19505	107.32	91.87	93.54	91.87–107.32
58	7.87	Galloyl-paeoniflorin	C_30_H_31_O_15_^−^	631.16919	95.80	50.35	77.00	50.35–95.80
∑ Total recovery of terpenoids					80.09	90.25	63.56	63.56–90.25
∑∑ Total recovery of detected bioactive compounds					81.91	72.47	71.75	71.75–81.91

GID: digestion in the gastrointestinal tract; DPT: digested of *Paeonia tenuifola* tea; DPP: digested of *Paeonia peregrina* tea; DPO: digested of *Paeonia officinallis* tea. ** Compound identified also in digested sample. The recovery of total and individual compounds was calculated as the ratio of areas before and after *in vitro* GID. “-” not an identified compound.

## Data Availability

Data are contained within the article and Supplementary Materials.

## Supplementary Materials

The following supporting information can be downloaded at: https://www.mdpi.com/article/10.3390/ph17040518/s1, Figure S1 (Supp. 1): Preliminary screening of factor level’s impact (locality and extraction procedure) on the total polyphenolic concentration (TPC) of *Paeonia tenuifolia* L; Figure S2 (Supp. 2): ATR-FTIR spectra of the root extracts of *Paeonia tenuifolia* L. (PT), *Paeonia peregrina* Mill. (PP), and *Paeonia officinalis* L. (PO); UAE—ultrasound-assisted extraction; Figure S3 (Supp. 2): ATR-FTIR spectra of the root extracts of *Paeonia tenuifolia* L. (PT), *Paeonia peregrina* Mill. (PP), and *Paeonia officinalis* L. (PO); M—maceration; Table S1 (Supp. 3): Full factorial design for screening of factors’ influence on total polyphenolic concentration (TPC) of root extracts of *Paeonia tenuifolia* L., *Paeonia peregrina* Mill., and *Paeonia officinalis* L., with the measured and predicted values; Table S2 (Supp. 3): Characterisation of detected bioactive compounds in selected peony root teas before and after *in vitro* GID, using UHPLC-QToF-MS. Target compounds, expected retention time (RT), base peak, molecular formula, calculated mass, exact mass and MS^2^ fragments are presented.
