# Supplementary material for: Phytochemical Analysis, Biological Activities, and Molecular Docking Studies of Root Extracts from Paeonia Species in Serbia
Source: Pharmaceuticals (Basel). 2024 Apr 17;17(4):518. doi: 10.3390/ph17040518 (PMC11054981; doi:10.3390/ph17040518)
Supplement: Supplementary file 1 [file pharmaceuticals-17-00518-s001.zip › pharmaceuticals-2918694-supplementary.pdf]

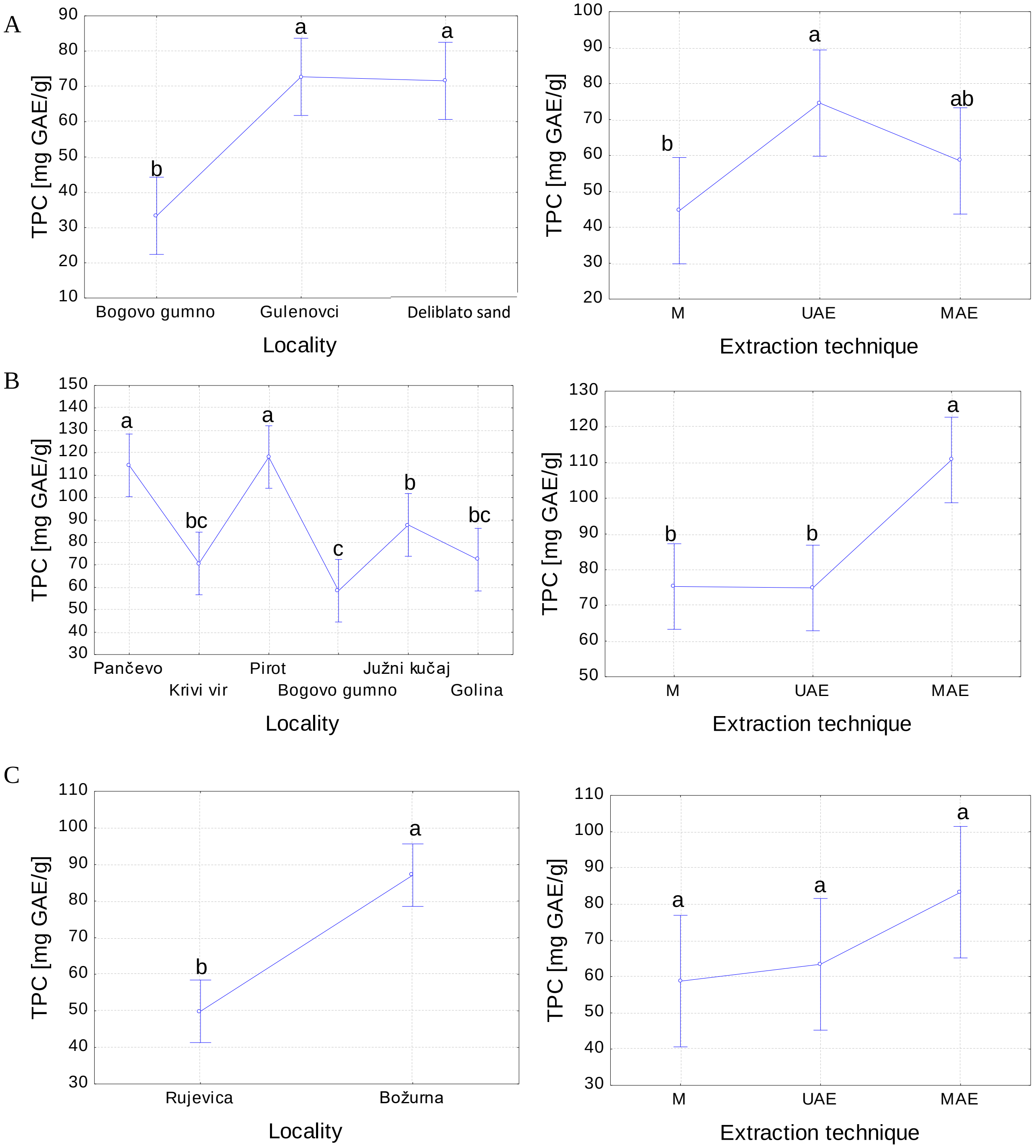

Figure S1. Preliminary screening of factor level's impact (locality and extraction procedure) on the total polyphenolic concentration (TPC) of *Paeonia tenuifolia* L. **(A)**, *Paeonia peregrina* Mill. **(B)**, and *Paeonia officinalis* L. **(C)** root extracts; GAE, gallic acid equivalents; MAE, microwave-assisted extraction; UAE, ultrasound-assisted extraction; M, maceration; values with different letters showed statistically significant difference (analysis of variance - one way ANOVA followed by Duncan's *post hoc* test, $p < 0.05$; $n = 3$).

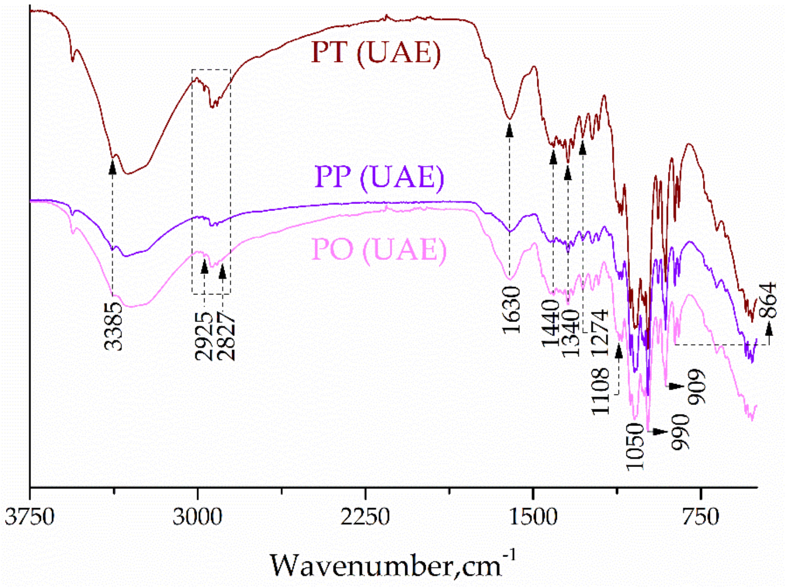

Figure S2. ATR-FTIR spectra of the root extracts of *Paeonia tenuifolia L.* (PT), *Paeonia peregrina* Mill. (PP), and *Paeonia officinalis* L. (PO); UAE – ultrasound-assisted extraction

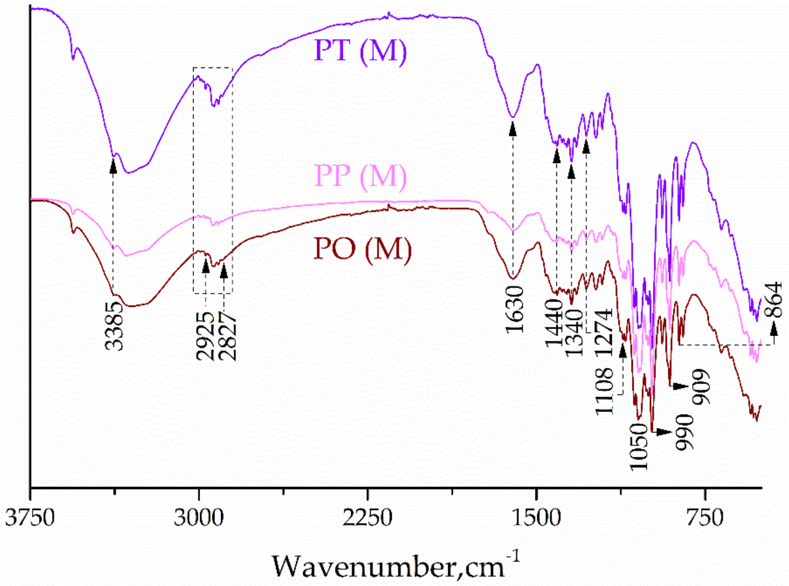

Figure S3. ATR-FTIR spectra of the root extracts of *Paeonia tenuifolia L.* (PT), *Paeonia peregrina* Mill. (PP), and *Paeonia officinalis* L. (PO); M – maceration

Table S1. Full factorial design for screening of factors' influence on total polyphenolic concentration (TPC) of root extracts of *Paeonia tenuifolia* L, *Paeonia peregrina* Mill., and *Paeonia officinalis* L., with the measured and predicted values.

**Species**	**Locality**	**Extraction procedure**	**TPC** [mg GAE*/mL]	
	Factor levels		Measured	Predicted
Paeonia tenuifolia L.	Gulenovci	MAE	77.2±2.6	76.8±2.1
	Gulenovci	UAE	87.9±1.4	87.7±2.1
	Deliblato	MAE	67.6±0.7	67.5±2.0
	**Deliblato sand**	**UAE**	**99.6±0.2**	**99.6±1.9**
Paeonia peregrina Mill.	**Pirot**	**MAE**	**161.2±2.2**	**161.1±3.5**
	Pirot	M	99.9±1.8	99.7±3.2
	Pančevo	MAE	143.7±2.6	143.2±3.1
	Pančevo	M	91.1±2.6	90.9±3.4
Paeonia officinalis L.	Rujevica	MAE	65.6±1.0	65.5±2.1
	Rujevica	M	35.0±1.5	35.0±1.9
	**Božurna**	**MAE**	**101.2±2.8**	**101.1±1.8**
	Božurna	M	82.6±1.6	82.5±2.0

*GAE: gallic acid equivalent; Maceration (M); Microwave-assisted extraction (MAE); Ultrasound-assisted extraction (UAE)

Table S2. Characterisation of detected bioactive compounds in selected *Peonie* root teas before and after *in vitro* GID, using UHPLC-QToF-MS. Target compounds, expected retention time (RT), base peak, molecular formula, calculated mass, exact mass and MS² fragments are presented.

No	RT	Compound name	Formula	Calculated mass	*m/z* exact mass	mDa	MS fragments
Phenolic acids and derivatives							
Gallic acid and derivatives							
1	1.14	**Gallic acid**	$C_7H_5O_5^-$	169.01370	169.01443	-0.73	107.01391(12), 123.00844(12), 124.01635(76), **125.02413(100)**, 126.02718(8)
2	4.24	**Methyl gallate**	$C_8H_7O_5^-$	183.02930	183.02825	1.05	106.00613(4), 123.00874(4), **124.01629(100)**, 125.02(8)
3	1.75	**Gallic acid derivative**	$C_{10}H_{11}O_7^-$	243.05050	243.05244	-1.94	107.01344(9), 123.00887(8), **124.01625(100)**, 125.02312(29), 168.00758(2), **169.01439(9)**
4	3.77	**Digallic acid isomer I**	$C_{14}H_9O_9^-$	321.02470	321.02654	-1.84	**125.02403(100)**, 126.02838(6), **169.01388(65)**, 170.01809(5)
5	5.59	**Digallic acid isomer II**	$C_{14}H_9O_9^-$	321.02470	321.02743	-2.73	**125.02427(100)**, 126.02755(8), **169.01395(65)**, 170.01726(6)
6	0.90	**Gallic acid hexoside**	$C_{13}H_{15}O_{10}^-$	331.06650	331.06817	-1.67	107.01397(6), 123.00845(6), **124.01598(33)**, **125.02385(65)**, 168.00629(20), **169.01409(100)**
7	8.02	**Methyl digallate**	$C_{15}H_{11}O_9^-$	335.04030	335.04267	-2.37	**124.01643(46)**, 125.02152(5), 168.00576(9), 169.01352(5), **183.03013(100)**, 184.03288(10), **335.11527(2)**
8	1.75	**Methyl gallic acid hexoside**	$C_{14}H_{17}O_{10}^-$	345.08220	345.08617	-3.97	107.01447(30), 123.00877(60), 124.01631(44), **125.02357(46)**, 168.0063(27), **169.01243(53)**, **183.02919(100)**
9	7.34	**Galloyl-vanilloyl-rhamoside**	$C_{21}H_{21}O_{12}^-$	465.10330	465.10780	-4.5	**125.02435(100)**, 151.03506(6), **152.0114(25)**, 217.0503(13), 259.06067(24), 275.05562(26), 285.04029(44), 437.11006(24), 465.10427(10)
10	1.00	**Gallic acid dihexoside**	$C_{19}H_{25}O_{15}^-$	493.11930	493.12260	-3.30	125.02384(18), 151.00411(18), **169.01422(95)**, 170.01728(9), 211.02467(8), 223.02426(8), 241.0353(11), 271.04636(31), 283.04582(33), **313.0571(100)**, 314.06049(17), **331.06745(13)**, 493.12092(43)
11	5.93	**Galloyl-HHDP-hexose isomer II**	$C_{27}H_{21}O_{18}^-$	633.07280	633.07382	-1.02	169.01383(6), 275.01953(14), **300.99944(100)**, 302.00219(18), 303.00463(3), 313.05586(2), 463.05151(9), 483.07984(2), 633.07382(38)
12	2.89	**Galloyl-HHDP-hexose isomer I**	$C_{27}H_{21}O_{18}^-$	633.07280	633.07577	-2.97	169.01297(8), 275.02003(18), 276.02319(3), **300.99915(100)**, 302.00128(18), 463.05188(10), 481.06299(11), 633.07577(16)
13	6.80	**Trigalloyl-hexoside**	$C_{27}H_{23}O_{18}^-$	635.08840	635.09260	-4.20	125.02319(5), **169.01359(33)**, 170.01952(3), 193.01332(3), 211.02546(3), 223.02472(2), **313.05705(31)**, **465.06726(100)**, 466.07048(30), 467.07544(6), 483.07466(5), 635.08707(2)
14	8.15	**Digalloyl-HHDP-protoquercitol**	$C_{34}H_{27}O_{21}^+$	771.10450	771.10856	-4.06	**125.02395(2)**, 127.03941(4), **153.01948(100)**, 154.02237(10), 233.04528(2), 279.05108(3), 305.02931(1)
15	7.55	**Tetragalloyl-hexoside**	$C_{34}H_{27}O_{22}^-$	787.09940	787.10066	-1.26	125.02408(4), **169.01365(35)**, 295.04601(11), **313.05651(5)**, 447.05715(10), 465.06804(24), **617.07864(45)**, 618.08311(16), 635.08994(35), 636.09267(11), **787.10066(100)**, 788.10351(47)
16	7.95	**Pentagalloyl-hexoside**	$C_{41}H_{31}O_{26}^-$	939.11040	939.11237	-1.97	**169.01396(12)**, 431.06149(5), 447.05781(6), 599.06893(5), 601.08286(5), 617.0801(15), **769.09121(78)**, 770.09408(35), 787.10071(9), **939.11237(100)**, 940.1152(57)
Ellagic acid and derivatives							
17	7.68	**Ellagic acid**	$C_{14}H_5O_8^-$	300.99840	301.00097	-2.57	125.02388(6), 145.02898(9), 157.02928(4), 173.02432(9), 185.02407(11), 201.01864(12), 217.01543(4), 226.99896(5), 229.01379(18), 245.00886(11), 257.00902(7), 283.99642(18), 299.9908(18), **300.99841(100)**
18	8.42	**Methyl ellagic acid**	$C_{15}H_7O_8^-$	315.01410	315.01710	-3.00	216.0064(5), 228.00601(1), 298.98408(1), **299.99116(100)**, 300.99505(18)
19	9.57	**Dimethyl ellagic acid**	$C_{16}H_9O_8^-$	329.02970	329.03194	-2.24	270.98904(73), 271.99193(12), 283.99568(1), 285.00451(2), **298.98302(100)**, **299.98723(18)**, 300.98859(3), **314.00732(17)**, 315.01067(3)
20	10.85	**Trimethyl ellagic acid**	$C_{17}H_{11}O_8^-$	343.04540	343.04668	-1.28	269.9802(17), 270.98487(2), 285.00418(22), 286.0079(4), **297.97598(65)**, 298.97948(13), 299.98374(2), **312.99909(100)**, 314.00247(19), 315.00438(3), **328.02256(10)**, 329.0265(2)
21	6.87	**Ellagic acid hexoside**	$C_{20}H_{15}O_{13}^-$	463.05130	463.05601	-4.71	299.99183(70), **300.99829(100)**, 302.00177(17), 463.05277(14)
22	7.55	**Methyl ellagic acid hexoside**	$C_{21}H_{17}O_{13}^-$	477.06690	477.07058	-3.68	121.02928(15), 137.0228(2), 245.08164(3), 270.98938(3), 289.071(12), 298.98325(14), **299.99098(43)**, 300.99478(10), 314.00647(22), **315.01462(100)**, 316.01712(19), 317.01938(3), 477.06888(11)

Other phenolic acid and its glycosides							
23	3.09	**Hydroxybenzoic acid**	$C_7H_5O_3^-$	137.02390	137.02422	-0.32	/
24	1.88	**Dihydroxybenzoic acid isomer I**	$C_7H_5O_4^-$	153.01880	153.01946	-0.66	107.01064(10), **108.01956(100)**, 109.02881(82)
25	5.12	**Dihydroxybenzoic acid isomer II**	$C_7H_5O_4^-$	153.01880	153.01911	-0.31	**107.0123(100)**, 109.02803(17)
26	2.36	**Dihydroxybenzoic acid hexoside**	$C_{13}H_{15}O_9^-$	315.07160	315.07520	-3.60	108.02123(13), **109.02917(100)**, 110.03293(7), 152.01062(21), **153.0189(57)**, 315.0696(2)
27	2.96	**Vanillic acid hexoside**	$C_{14}H_{17}O_9^-$	329.08730	329.08958	-2.28	107.01288(9), 108.02132(16), 121.02787(18), 122.03681(20), **123.04492(100)**, 151.0045(19), 152.01052(23), 166.02598(19), **167.03379(49)**
Flavonoids and derivatives							
Flavan-3-ols and procyanidins							
28	5.29	**Catechin**	$C_{15}H_{13}O_6^-$	289.07120	289.07260	-1.40	109.02935(99), 121.02937(29), **123.04498(100)**, 125.02435(44), 137.02437(26), 138.03108(11), 149.02482(16), 151.03982(31), 159.04507(11), 161.0587(15), 164.01307(12), 187.04006(12), 203.07071(21), 221.08194(13)
29	6.63	**Epicatechin**	$C_{15}H_{13}O_6^-$	289.07120	289.07259	-1.39	109.02954(94), 121.02973(28), 122.03668(17), **123.04502(100)**, 125.02442(41), 137.02444(30), 149.025(15), 151.03999(32), 159.04542(11), 161.05922(14), 164.01304(13), 187.03949(11), 188.04753(11), 203.07075(21), 221.08248(13)
30	6.54	**Methyl epigallocatechin**	$C_{16}H_{15}O_7^-$	319.08180	319.08293	-1.13	121.02877(19), **125.02406(100)**, 126.0278(9), 137.02401(16), 149.02384(5), 161.02384(32), 162.02964(9), 164.01128(46), 165.01799(25), 175.04116(3), 203.03594(3)
31	7.68	**Epicatechin-gallate**	$C_{22}H_{17}O_{10}^-$	441.08220	441.08941	-7.21	109.02933(6), 124.01656(12), 125.02396(47), 137.02411(7), 151.0386(7), **169.01398(100)**, 179.03487(4), 193.01296(4), 203.07093(7), 205.05023(8), 245.08159(16), **289.07096(26)**, 290.07692(4)
32	4.18	**Catechin hexoside**	$C_{21}H_{23}O_{11}^-$	451.12400	451.12584	-1.84	109.02893(13), 123.04501(8), 125.02392(17), 137.02506(11), 149.02424(6), 151.04064(9), 165.01992(7), 179.03456(11), 203.07116(17), 221.08245(4), 245.08188(47), **289.07176(100)**, 290.0749(20)
33	5.73	**Epicatechin-hexoside**	$C_{21}H_{25}O_{11}^+$	453.13970	453.14384	-4.14	123.04507(47), 124.04734(5), 127.03965(3), **139.04014(100)**, 147.04498(15), 151.04276(2), 163.04196(3), 165.0554(19), 179.07106(2), 207.06507(9), 273.07562(3), **291.08474(3)**
34	4.51	**B type procyanidin dimer isomer I**	$C_{30}H_{25}O_{12}^-$	577.13515	577.13795	-2.80	125.02456(61), 161.02495(20), 245.07988(22), **289.07221(100)**, 339.08737(9), 407.07872(69)
35	6.33	**B type procyanidin dimer isomer II**	$C_{30}H_{25}O_{12}^-$	577.13515	577.13723	-2.08	125.02419(69), 161.02427(21), 245.08000(24), **289.07174(100)**, 339.08743(9), 407.07850(82)
36	6.63	**Chalcan flavan-3-ols dimer isomer II**	$C_{30}H_{27}O_{12}^-$	579.15030	579.15511	-4.81	109.02957(7), 123.04512(4), 125.02411(9), 137.02398(7), 179.03478(9), 203.07122(10), 205.05053(10), 245.08177(34), 246.08548(6), 249.08046(11), **289.07228(100)**, 290.0755(19)
37	5.29	**Chalcan flavan-3-ol dimer isomer I**	$C_{30}H_{27}O_{12}^-$	579.15030	579.15287	-2.57	109.02947(6), 123.04492(3), 125.02454(9), 137.02442(6), 151.03967(4), 161.05986(2), 165.01921(4), 179.03486(8), 203.07166(9), 205.05112(9), 245.08274(32), **289.07199(100)**, 290.07574(19)
38	6.19	**Methyl B type prodelphinidin**	$C_{31}H_{27}O_{13}^-$	607.14572	607.14904	-3.33	**125.02417(100)**, 161.02352(28), 243.03135(27), 261.03951(13), **287.05543(33)**, 405.06185(11)
39	6.02	**Procyanidin trimer B type isomer I**	$C_{45}H_{37}O_{18}^-$	865.19800	865.19890	-0.90	125.02426(79), 243.03073(23), **287.05641(100)**, 289.07077(69), 407.07849(70), 413.08807(32), 425.08796(46), 449.08803(26), 451.10294(33), 575.11956(34), **577.13507(58)**, 695.1413(43), 713.15203(24), 865.1989(35), 866.20364(24)
40	6.93	**Procyanidin trimer B type isomer II**	$C_{45}H_{37}O_{18}^-$	865.19800	865.19733	0.67	125.02404(99), 161.02504(26), **287.05528(100)**, 289.07164(66), 407.07683(77), 413.08747(39), 425.08753(58), 449.08883(29), 451.10247(39), 575.11963(44), **577.13561(58)**, 695.14215(38), 713.14768(31), 865.19733(32), 866.20019(27)
Other detected flavonoids							
41	10.1	**Kaempferol**	$C_{15}H_9O_6^-$	285.03990	285.04097	-1.07	107.01327(8), 143.05034(7), 145.03047(7), 151.00397(5), 157.06081(6), 159.04471(13), 185.06073(13), 187.0395(11), 211.04083(7), 214.02871(6), 229.05056(11), 239.03424(9), **285.03994(100)**, 286.04366(21)
42	9.91	**Naringenin**	$C_{15}H_{11}O_5^-$	271.06060	271.06201	-1.41	107.01443(26), **119.0502(100)**, 123.0446(38), 125.02393(7), **151.00323(30)**, 152.00829(3), 161.05956(3), 165.01893(3), 167.03416(18), 177.01894(4), 185.0627(2), 187.0508(3), 189.05406(7)

43	8.49	**Phloridzin**	$C_{21}H_{23}O_{10}^{-}$	435.12910	435.12768	1.42	119.0504(4), 123.04442(12), 125.02413(17), **167.03483(100)**, 168.0311(13), 179.03475(12), **273.07683(60)**, 274.07959(12)
Peonia root terpenoids							
44	9.97	**Nor-paeonilactone**	$C_{9}H_{15}O_{2}^{+}$	155.10720	155.10880	-1.60	103.05831(35), 105.06789(21), 107.08606(29), 110.98355(21), 111.04547(28), 115.05664(71), 116.97745(87), 127.05882(41), **128.06025(100)**, 129.07032(34), 131.07962(24), 144.09357(22), 156.07957(20), 157.07408(22), 158.09685(25)
45	6.40	**Paeoveitol D**	$C_{10}H_{11}O_{3}^{+}$	179.07080	179.07217	-1.37	**103.05503(100)**, 105.03189(10), **105.07108(82)**, 106.07323(22), 107.05239(28), 108.05868(26), 115.05622(12), 117.06879(14), 118.04748(20), 121.06358(13), 123.0432(17), 133.06625(15), 137.06064(14), 147.07017(11)
46	6.93	**Paeonisothujone**	$C_{10}H_{15}O_{3}^{+}$	183.10210	183.10219	-0.09	103.05502(35), 104.06195(21), 105.07028(23), 107.04943(11), 109.06526(14), 113.06101(18), 115.05534(20), 117.07058(64), 118.07414(10), **119.08661(100)**, 120.09063(12), 121.06599(21), 132.05737(20), 147.08113(11)
47	6.60	**Paeonilactone B**	$C_{10}H_{13}O_{4}^{+}$	197.08140	197.08347	-2.07	108.02146(35), 119.04954(4), 123.04508(80), 124.04883(7), 136.01797(4), **151.03983(100)**, 152.04355(12)
48	3.50	**Paeonilactone A**	$C_{10}H_{15}O_{4}^{+}$	199.09700	199.09852	-1.52	105.07061(46), **107.05018(100)**, 108.05485(14), 109.06747(21), 111.083(12), 115.05495(57), 117.06995(65), 119.08594(15), 123.08093(10), 125.0969(10), 135.08133(94)
49	2.62	**9-Hydroxypaeonilactone A**	$C_{10}H_{15}O_{5}^{+}$	215.09190	215.09411	-2.21	109.06581(83), 110.06978(10), 125.06083(6), 126.09247(6), **127.07618(100)**, 128.07962(11), 137.06052(32), 138.06538(4), 155.07111(47), 172.07668(6), 198.11391(4)
50	10.3	**Paeoniflorigenone**	$C_{17}H_{19}O_{6}^{+}$	319.11762	319.12118	-3.57	**105.03426(100)**, 133.06563(10), 137.06054(18), 151.07599(11), 179.07131(19)
51	5.82	**Oxypaeoniflorin**	$C_{23}H_{27}O_{12}^{-}$	495.15030	495.15174	-1.44	**137.024(100)**, 138.02759(8), 151.041(2), 165.05549(7), 177.05571(5), 195.06596(4), 281.06702(4), 333.09801(3), 345.11916(2), 465.14178(3), 495.15188(9)
52	8.90	**Mudanpioside D**	$C_{24}H_{29}O_{12}^{-}$	509.16590	509.17478	-8.88	**121.02881(100)**, 122.03155(13), 123.04305(6), 152.0098(7), 153.00127(6), 167.03275(14), 169.01321(9), 227.13876(6), 333.02808(8), 347.17663(9), 465.20628(7), 509.17994(6)
53	7.07	**Paeoniflorin+HCOOH**	$C_{24}H_{29}O_{13}^{-}$	525.16080	525.16398	-3.18	113.02443(4), **121.02931(100)**, 122.0331(9), 165.05583(10), 177.05564(2), 327.10898(4)
54	7.75	**Albiflorin+HCOOH**	$C_{24}H_{29}O_{13}^{-}$	525.16080	525.16361	-2.81	113.02422(4), **121.02911(100)**, 122.03275(9), 165.05557(9), 177.05555(2), 327.10913(3)
55	2.59	**Galloyl desbenzoyl paeoniflorin**	$C_{23}H_{27}O_{14}^{-}$	527.14010	527.14182	-1.72	123.0082(5), 124.01619(7), 125.02411(7), 151.00367(7), 165.055(13), 168.00677(5), 169.01362(67), 271.09703(6), 279.05119(7), 313.0562(15), 497.128(13), **527.14182(100)**, 528.14457(32)
56	10.1	**Benzoyl paeoniflorin**	$C_{30}H_{33}O_{12}^{+}$	585.19720	585.20263	-5.43	**105.03473(100)**, 106.0376(10), 109.02798(5), 121.06425(3), 123.04621(5), 127.04(4), 133.06555(9), 151.07716(10), 153.01581(3), 161.05998(3), 179.06945(4), 197.08179(9), 249.07663(10), 267.08642(3)
57	9.91	**Mudanpioside J**	$C_{31}H_{33}O_{14}^{-}$	629.18700	629.19505	-8.05	**121.02932(100)**, 122.0328(9), 135.04493(2), 165.05529(5), 177.05463(2), 431.13521(1)
58	7.87	**Galloyl-paeoniflorin**	$C_{30}H_{31}O_{15}^{-}$	631.16684	631.16919	-2.34	169.01421(34), 271.04647(59), 313.05735(62), 491.12065(45), 613.15807(59), **631.16841(100)**
